# *In Situ* Time-Resolved X-ray Absorption Spectroscopy Unveils Partial Re-Oxidation of Tellurium Cluster for Prolonged Lifespan in Hydrogen Evolution

**DOI:** 10.1021/jacs.5c00167

**Published:** 2025-04-15

**Authors:** Kanglei Pang, Chang Long, Yu Zhang, Miao Zhang, Jian Chang, Yong-Lei Wang, Hao Zhang, Rongying Liu, Sadaf Saeedi Garakani, Özlem Uguz Neli, Jiayin Yuan

**Affiliations:** † Department of Chemistry, Stockholm University, Stockholm 10691, Sweden; ‡ Institute of Fundamental and Frontier Sciences, University of Electronic Science and Technology of China, Chengdu, Sichuan 611731, PR China

## Abstract

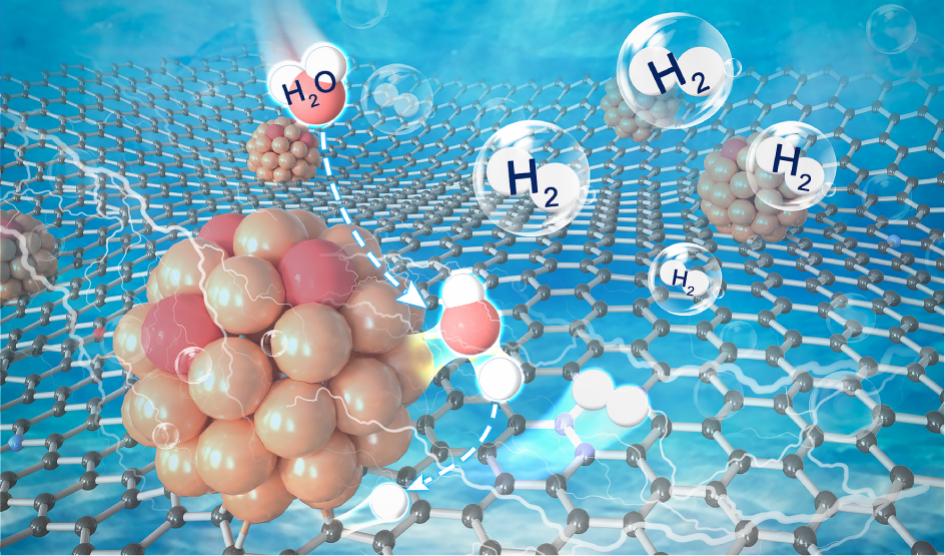

Efficient and long-lasting electrocatalysts are one of the key factors in determining their large-scale commercial viability. Although the fundamentals of deactivation and regeneration of electrocatalysts are crucial for understanding and sustaining durable activity, little has been conducted on metalloids compared to metal-derived ones. Herein, by virtue of *in situ* seconds-resolved X-ray absorption spectroscopy, we discovered the chemical evolution during the deactivation-regeneration cycles of tellurium clusters supported by nitrogen-doped carbon (termed Te-ACs@NC) as a high-performance electrocatalyst in the hydrogen evolution reaction (HER). Through *in situ* electrochemical reduction, Te-ACs@NC, which had been deactivated due to surface phase transitions in a previous HER process, was reactivated and regenerated for the next run, where partially oxidized Te was found, surprisingly, to perform better than its nonoxidized state. After 10 consecutive deactivation-regeneration cycles over 480 h, the Te-ACs@NC retained 85% of its initial catalytic activity. Theoretical studies suggest that local oxidation modulates the electronic distribution within individual Te clusters to optimize the adsorption energy of water molecules and reduce dissociation energy. This study provides fundamental insights into the rarely explored metalloid cluster catalysts during deactivation and regeneration and will assist in the future design and development of supported catalysts with high activity and long durability.

## Introduction

Long durability and high activity are crucial performance indicators of electrocatalysts in the chemical industry.^1^ Improving the longevity of high-performance catalysts remains a major focus of the research community.^2,3^ Recently, atomically dispersed catalysts have attracted growing interest due to their distinctive catalytic activity.^4−7^ Very often, structure-sensitive atomically active configurations face the challenge of deactivation under prolonged operating conditions.^8^ For example, downsizing catalytically active components to clusters or single atoms lowers their coordination numbers and raises surface energy, rendering the catalyst–support interface more dominant.^9^ This makes them vulnerable to degradation, such as dissolution and Ostwald ripening, exacerbating the leaching risk of metal components or the agglomeration of single atom sites.^10,11^ Additionally, active and optimized structures of clusters or single atoms are more susceptible to environmental variations, leading to poisoning or potential-driven effects.^12^ These factors promote deactivation or unwanted structural rearrangements of surface active sites, limiting their durability and practical value.^13^

Several strategies have been proposed to address the stability issues of atomically dispersed catalysts in an electrochemical environment. These strategies include interfacial engineering to regulate the geometric and electronic structures, interfacial bonding in heterostructure electrocatalysts, and more.^14^ For instance, strengthening metal–support and cluster–cluster interactions will enhance both reaction kinetics and catalyst durability.^15−17^ An alternative approach involves the introduction of protective agents, such as metal oxide supports or surfactants, or the employment of spatially confined encapsulation layers (e.g., one-dimensional tubular or three-dimensional porous materials) to build multilevel structures.^18,19^ These efforts selectively reduce the adsorption of poisonous substances, stabilize active sites, and shield catalysts from environmental variables.^20^ However, increasing longevity often comes at the cost of reduced catalytically active sites and limited mass transfer, ultimately sacrificing catalytic activity. Furthermore, these catalysts constantly face increasing deactivation risks upon prolonged operation, necessitating replacement.^21^ This underscores the need for periodic and continuous regeneration of deactivated catalysts.^22^

Conventional X-ray absorption spectroscopy (XAS) typically requires about 10 min per spectrum. Note that both deactivation and regeneration of catalysts are relatively rapid processes, particularly when the dynamic restructuring of surface active sites is concerned, which can stabilize within minutes.^23^ In this context, time-resolved identification of the chemical state of the catalyst surface is essential to understand its behavior at each time point under relevant reaction conditions.^24,25^ In particular, on a time scale of merely a few seconds, it is critical to identify the dominant factors responsible for the deactivation and regeneration of catalysts. Time-resolved XAS is a quickly advancing analytic technique and has been considered pivotal in addressing these scientific challenges. *In situ* time-resolved XAS with seconds resolution have been used to probe dynamic geometric and electronic changes in working catalysts.^26−30^ By collecting data within seconds—such as X-ray absorption near-edge structure (XANES) and extended X-ray absorption fine structure (EXAFS)—this technique seizes real-time views of the chemical state of catalysts and surface adsorbates, approaching the genuine status of dynamic deactivation and regeneration processes that are otherwise hard to access.

Herein, we present a time-resolved XAS investigation of highly dispersed cluster-type Te-ACs@NC catalysts for HER. The freshly synthesized Te-ACs@NC, upon aerobic storage, was found to be trapped in a partially oxidized stable state, and full oxidation occurred during extended HER operation. Under an applied reduction potential, the deactivated fully oxidized Te-ACs@NC, which had undergone a phase transition, was reduced and reactivated for catalysis, completing one oxidation-deactivation-reduction-regeneration loop. Even after 10 cycles, its catalytic performance drops by only 15%, demonstrating excellent durability. Notably, Te, as a semimetal, competes with the state-of-the-art Pt catalyst in terms of HER activity. *In situ* seconds-resolved XAS characterization revealed that during one-day exposure to air, the electroreductively regenerated Te-ACs@NC was subsequently reoxidized partially into a metastable state. Density functional theory (DFT) calculations suggested that local oxidation, which usually poisons metal catalysts, optimized the electronic distribution in Te-ACs@NC. It enhanced water adsorption and lowered the energy barrier for H_2_O dissociation into *H and *OH. This work sheds light on the fundamental understanding and rational design of metalloid catalysts with high activity and long-life stability in hydrogen electrocatalysis and potentially other catalyzes.

## Results

### Physical and Chemical Nature of the Te-ACs@NC Catalysts

The catalytic system of interest consists of Te-based clusters supported by a nitrogen-doped carbon material. Te serves as a cheaper substitute for Pt, as it costs <1% of Pt.^31^ Te-ACs@NC was was synthesized in three straightforward steps (Figure S1). First, surface-quaternized cellulose nanofibrils (CNFs) were extracted from wood pulp fibers by chemically reacting hydroxyl groups with glycidyltrimethylammonium chloride to form quaternary ammonium chloride ion pairs on the CNFs. The cation density on the CNF surface, which later determined the N content in the carbonized product, was controlled by the mass ratio of glycidyltrimethylammonium chloride to pulp during synthesis. CNFs with a high surface cation density of 1615 μeq g^–1^ were produced in this study Second, cationic CNFs were anion-exchanged with potassium tellurite (K_2_TeO_3_) to replace chloride anions. To stress, in this study, K_2_TeO_3_ was mixed with cationic CNFs in three mass ratios of 1:100, 2:100, and 10:100 to prepare three Te-containing cellulosic aerogels, termed Te@CNFs-*x*, where *x* represents the relative mass percentage (1, 2, and 10, respectively). Third, Te-ACs@NC was produced at 900 °C under argon by pyrolyzing Te@CNFs-1, the aerogel with the lowest tellurite content. When pyrolyzing Te@CNFs-2 and Te@CNFs-10 aerogels with higher tellurite content, Te nanoparticles of different sizes were produced on nitrogen-doped porous carbon, and these were termed Te-NPs-1@NC and Te-NPs-2@NC, respectively. As a control, a Te-free carbon sample, termed NC900, was prepared via the same procedure, except without the addition of potassium tellurite.

**Figure 1:**
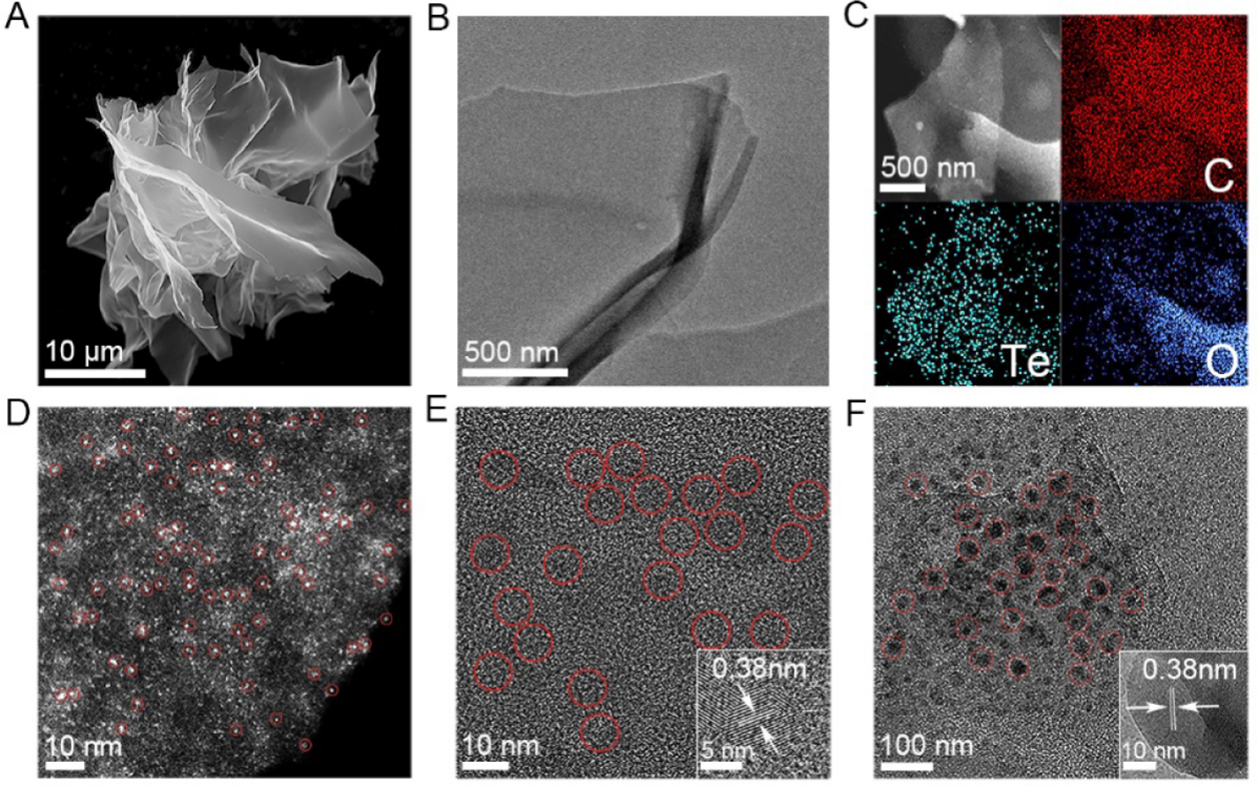
Morphological characterization. (A) SEM image, (B) TEM image, and (C) elemental mapping of Te-ACs@NC; (D) HAADF-STEM image of Te-ACs@NC; TEM and HRTEM (inset) images of Te-NPs-1@NC (E); and Te-NPs-2@NC (F).

Raman spectroscopy and X-ray diffraction (XRD) analysis detected the structural evolution of Te clusters/particles from CNF aerogels as well as the graphitization process (Figure S2). The XRD diagrams of Te-ACs@NC confirm the absence of the crystalline Te phase. In the Raman spectrum, the vibrational bands at 116 and 138 cm^–1^ correspond to the A1 and E2 phonon modes, respectively, suggesting chain expansion within the basal plane and asymmetric stretching of Te atoms in helical chains.^32−34^ Additionally, two distinct carbon-related bands were observed at 1588 cm^–1^ (G band) and 1340 cm^–1^ (D band) (*I*_D_/*I*_G_ = 0.98), indicating a high degree of graphitization at 900 °C. The morphology and microstructure of Te-ACs@NC were visualized using scanning electron microscopy (SEM) and transmission electron microscopy (TEM, Figure 1A,B), and compared with Te-NPs-1@NC, Te-NPs-2@NC, and NC900 (Figures 1E,F,S3, and S4). The results reveals that all synthesized carbocatalysts exhibited thin, sheet-based card-house-like structures. The presence and uniform distribution of Te and O elements within the carbon matrix of Te-ACs@NC were confirmed by STEM-coupled energy-dispersive X-ray spectroscopy (EDS) (Figure 1C). Aberration-corrected high-angle annular dark-field scanning transmission electron microscopy (HAADF-STEM) visualized an even distribution of Te clusters in Te-ACs@NC, evidenced as bright spots of ∼1 nm in the carbon framework, without Te (nano)particles being observed (Figures 1D and S5). The addition of more K_2_TeO_3_ to the synthesis of Te-NPs-1@NC and Te-NPs-2@NC formed Te nanoparticles of 10 ± 2 nm and 40 ± 6 nm, respectively (Figure S6). A lattice spacing of 0.38 ± 0.01 nm was observed in both high-resolution TEM (HRTEM) images (Figure 1E,F, inset), corresponding to the (100) plane of crystalline Te.^34,35^ Inductively coupled plasma optical emission spectrometry (ICP-OES) analysis measured the mass contents to be 1.90, 5.1, and 21.8 wt % in Te-ACs@NC, Te-NPs-1@NC, and Te-NPs-2@NC, respectively. The X-ray photoelectron spectroscopy (XPS) was used to study the chemical bonding of Te, C, and O species in the catalysts (Figure S7). As the Te concentration increased, the oxygen content and Te–O species rose as well due to the spontaneous oxidation of surface Te to TeO_2_ under atmospheric conditions (Figure S8 and Tables S1 and S2). Notably, the amounts of Te–Te, Te–O, and Te–C in Te-ACs@NC remained relatively stable after 24 h and eventually unchanged after 30 days, ending up with only a partial oxidation state under aerobic storage (Table S3).

**Figure 2:**
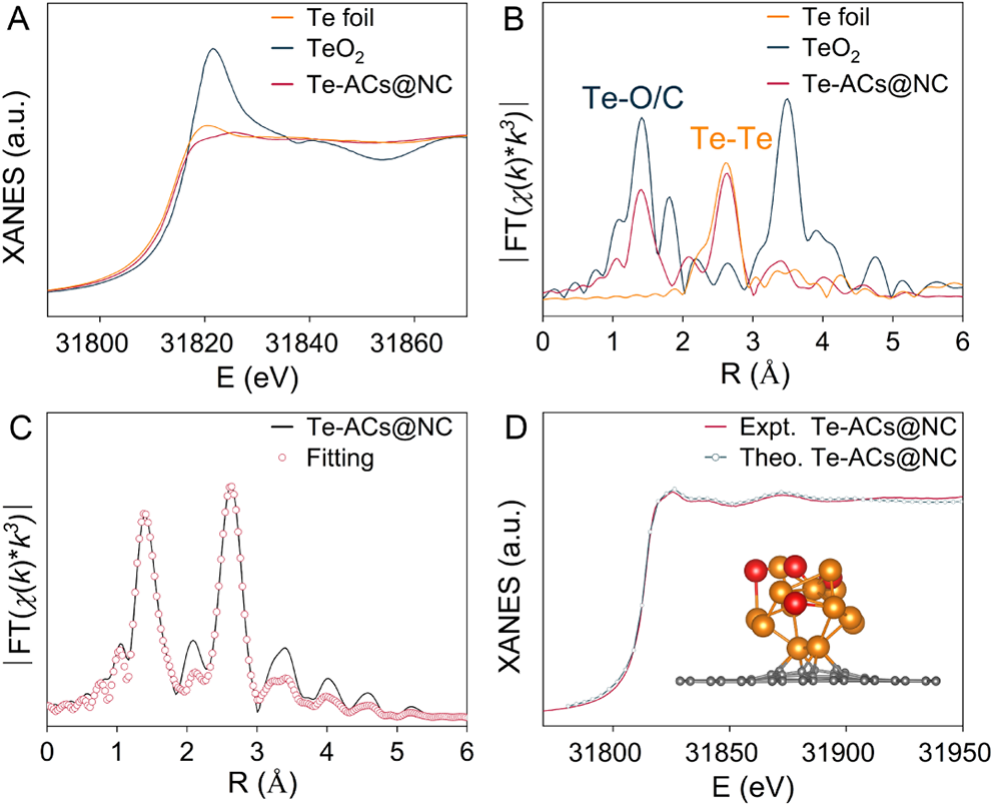
Structural characterization. (A) The experimental Te K-edge XANES spectra of Te-ACs@NC and the references (Te foil and TeO_2_); (B) FT k^3^-weighted Te K-edge EXAFS spectra of Te-ACs@NC and the references (distances not corrected for phase shift); (C) FT EXAFS fitting curves of Te-ACs@NC at the Te K-edge; (D) Comparison between the experimental K-edge XANES spectra and the theoretical spectra calculated from the inset model.

Synchrotron X-ray absorption spectroscopy (XAS) was employed to reveal the atomic structures of the Te clusters. In Figure 2A, the Te K-edge X-ray absorption near-edge structure (XANES) spectrum of Te-ACs@NC was compared with that of Te foil and TeO_2_ as references. The X-ray absorption edge (*E*_0_) of Te-ACs@NC lies between the Te foil and TeO_2_. The fitted oxidation state of Te in Te-ACs@NC from the K-edge XANES spectra is determined to be 1.32 (Figure S9). The Fourier-transformed k^3^-weighted extended X-ray absorption fine structure (EXAFS) spectra of Te-ACs@NC (Figure 2B) show a major peak at ∼2.60 and ∼1.41 Å (without phase shift Φ_ij_ correction), attributed to the distances between Te–Te and Te–O/C. Structural parameters of the Te K-edge were quantitatively extracted using least-squares EXAFS fitting. The fitted curves match the experimental spectra well (Figure 2C and Table S4). To identify the coordination environment of the Te cluster, we employed DFT calculations to establish plausible models with progressive degrees of oxidation. We then conducted Te K-edge XANES simulations for each model using the *ab initio* multiple scattering calculation FEFF 9.6.4.^36^ As presented in Figures 2D and S10, the calculated Te K-edge spectra closely replicate the experimental Te-ACs@NC spectra. The theoretical EXAFS spectra of Te-ACs@NC closely match the experimental data (Figure S11). We note that the muffin-tin approximation may not fully capture the surface and quantum size effects of subnanometer clusters. Nevertheless, FEFF9 provides a useful framework to compare the general trend of experimental EXAFS with theoretical predictions. We constructed a structural model that closely approximates the actual catalyst, in which the locally oxidized Te-ACs@NC consists of a Te_13_ cluster in partial oxidation with four oxygen atoms at its apex. Nonetheless, we acknowledge that due to the inherent limitations of current characterization methods, it is challenging to fully capture every structural detail—particularly in clusters with complex coordination environments and surface disorder.^37,38^

### Electrocatalytic Performance and Stability of Te-ACs@NC on HER

**Figure 3:**
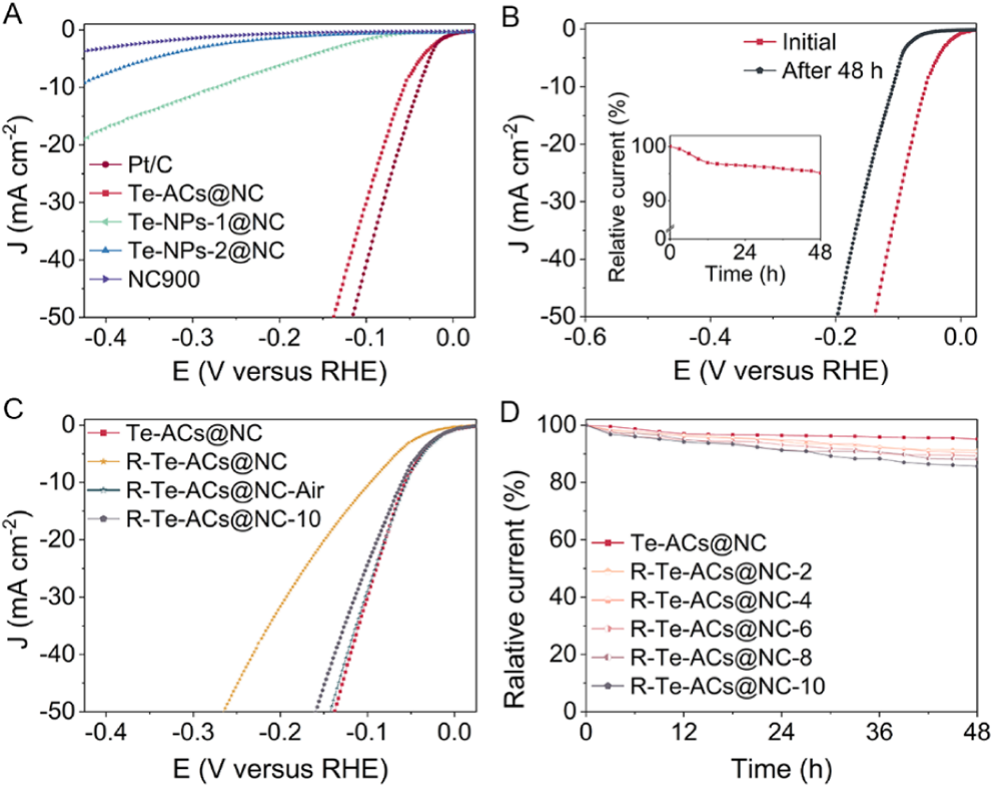
Electrochemical performance. (A) Representative HER polarization curves of Te-ACs@NC, Te-NPs-1@NC, Te-NPs-2@NC, NC900, and commercial Pt/C in 1.0 M aqueous KOH solution (see also Figure S11); (B) Chronopotentiometry curves of Te-ACs@NC over 48 h at a current density of 10 mA cm^–2^, along with LSV curves before and after the HER test; (C) Representative HER polarization curves of Te-ACs@NC, R-Te-NPs@NC (electrochemically reduced deactivated catalyst), R-Te-NPs@NC-Air (electrochemically reduced deactivated catalyst during one-day exposure to air), and R-Te-NPs@NC-10 (catalyst after 10 electrochemical reductions) in 1.0 M aqueous KOH solution; (D) Normalized current–time (*i*–*t*) chronoamperometric responses of Te-ACs@NC and R-Te-ACs@NC-*X* (*X* represents the number of times the catalyst was electrochemically reduced).

Linear sweep voltammetry (LSV) measurements in N_2_-saturated 1.0 M aqueous KOH solution were employed to evaluate the electrocatalytic HER activity of Te-ACs@NC. For comparison, the HER activities of Te-NPs-1@NC, Te-NPs-2@NC, commercial Pt/C (20 wt %), and NC900 catalysts were evaluated under identical conditions (Figures 3A and S12). As observed, the Te-ACs@NC catalyst exhibits superior HER catalytic activity with an onset potential of 5 mV (at 1 mA cm^–2^) in a current density of 10 mA cm^–2^ at a small overpotential of 56 mV (denoted as η_10_).^39^ The η_10_ values of Te-NPs-1@NC and Te-NPs-2@NC are 273 and 436 mV, respectively, impaired by the partial shielding of the Te nanoparticle surface due to adsorbed oxygen forming TeO_2_. Notably, the Tafel slope of Te-ACs@NC is 53 mV dec^–1^, suggesting the dominance of the Volmer–Heyrovsky pathway (Figure S13). Electrochemical impedance spectroscopy (EIS) and electrochemical surface area (ECSA) measurements were then conducted to assess the optimal HER capacity. Te-ACs@NC displays a low charge-transfer resistance (10.9 Ω), enhanced electrical conductivity (352 S cm^–1^), and high electrochemical surface area (265.5 cm^2^ mg^–1^) (Figures S14–S16). The enhanced charge transfer in Te-ACs@NC is attributed to the Te cluster loading. The inherent electrocatalytic efficiency of Te-ACs@NC was evaluated by calculating its turnover frequency (TOF, Figure S17). The Te-ACs@NC catalyst demonstrated high hydrogen production efficiency with TOF values of 0.61 and 2.30 H_2_ s^–1^ at overpotentials of 50 and 100 mV, respectively.

Long-term stability, one of the key operational characteristics, was evaluated using chronoamperometric testing at 10 mA cm^–2^, revealing a relative current loss of ∼5% after 48 h (termed Te-ACs@NC-48 h) (Figure 3B). Along this test, Te-ACs@NC electrodes showed activity loss with an ending η_10_ of 108 mV. Such loss is likely due to continued oxidation of the locally oxidized Te cluster during operation, leading to undesirable changes in the active site. To confirm this, we performed *in situ* electrochemical reduction by applying a constant reduction potential to the deactivated catalyst (termed R-Te-ACs@NC), and then formed the partially oxidized sample R-Te-ACs@NC-air during one-day exposure to air, possibly through route as well (Figure S18). As shown in Figure 3C, R-Te-ACs@NC exhibited lackluster catalytic activity (η_10_ = 97 mV), whereas R-Te-ACs@NC-air (η_10_ = 55 mV) delivered catalytic performance equivalent to Te-ACs@NC, and the structure of R-Te-ACs@NC-air remained essentially unchanged, as confirmed by HAADF-STEM and XRD results (Figure S19). Notably, we regenerated the catalyst multiple times and termed them R-Te-ACs@NC-*X*, where *X* is the number of regeneration cycles. Even after 10 cycles, R-Te-ACs@NC-10 retained 85% of its normalized current, demonstrating that the catalyst can extend its service life via multiple regenerations by *in situ* electrochemical reduction (Figure 3D).

### *In Situ* Time-Resolved XAS and Surface Characterization

To pinpoint the deactivation step during HER and the subsequent regeneration during *in situ* electrochemical reduction of Te-ACs@NC, we recorded both conventional *in situ* XAS and time-resolved XAS measurements to capture the chemical and structural evolution of the active centers on their respective time scales. Because catalyst deactivation typically unfolds over longer durations, while reactivation can occur more rapidly, we adapted our scanning protocols accordingly. Meanwhile, the well-defined structure of Te-ACs@NC enables precise *in situ* tracking of chemical environment changes, facilitating reliable performance correlations. At a potential corresponding to a current density of 10 mA cm^–2^ during HER, *in situ* XAS spectra were collected at 0, 1, 6 h, and every subsequent 6 h thereafter. During *in situ* electrochemical reduction, time-resolved XAS spectra were collected over 960 s, with each spectrum recorded at 10 s intervals. Additionally, following electrochemical reduction, *in situ* XAS spectra were collected every 3 h over 24 h. During both catalysis and electrochemical reduction, the external electrolyte was connected to the *in situ* XAS cell via a flow pump (Figure S20).

**Figure 4:**
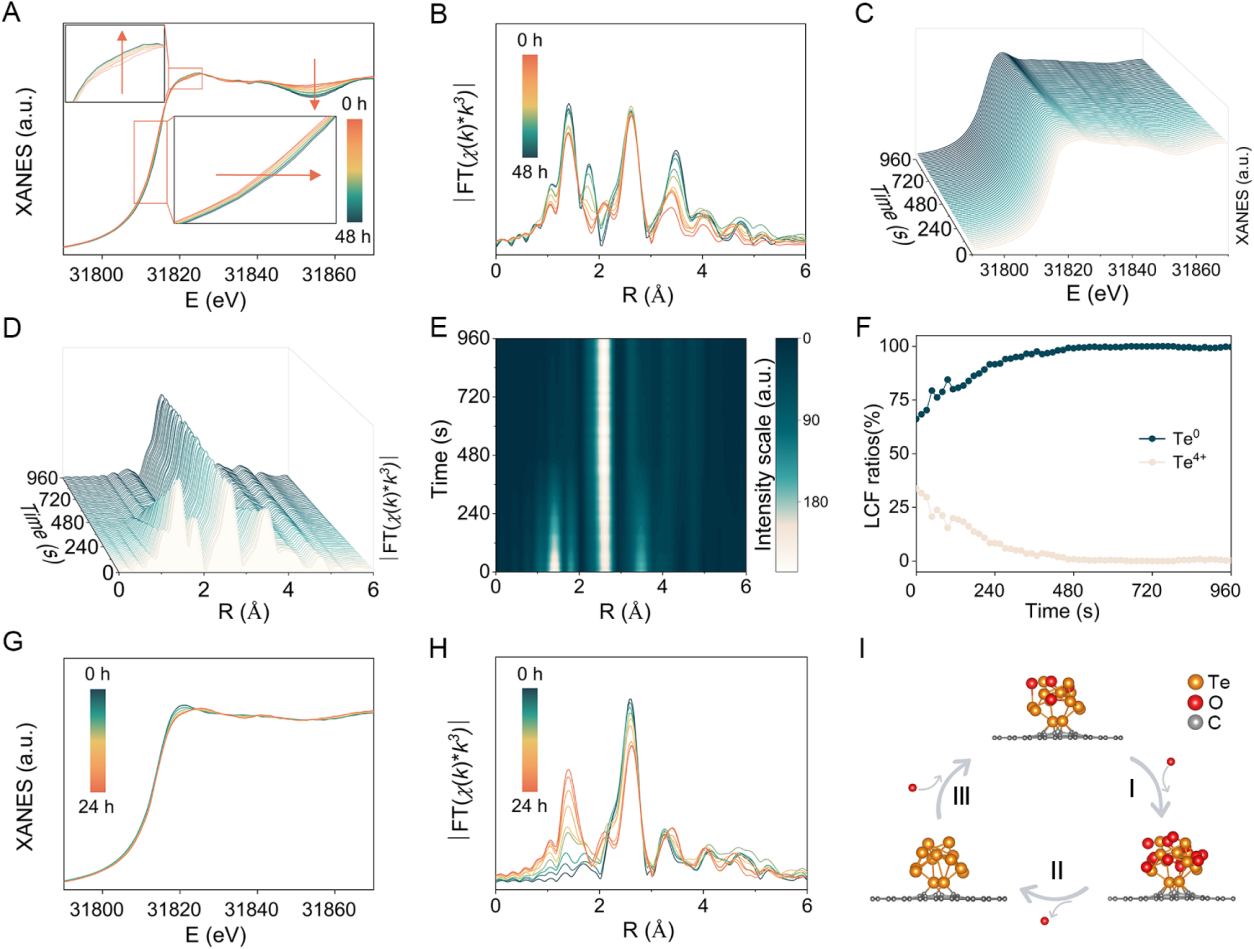
*In situ* and time-resolved XAS characterization of Te-ACs@NC. (A) *In situ* Te K-edge XANES spectra and (B) the corresponding EXAFS spectra recorded over 48 h of HER at 10 mA cm^–2^ in 1.0 M KOH; (C) *in situ* time-resolved Te K-edge XANES spectra and (D, E) the corresponding time-resolved EXAFS spectra under *in situ* electrochemical reduction at −1.1 V vs RHE; (F) LCF results of *in situ* time-dependent XANES spectra; (G) *in situ* Te K-edge XANES spectra; and (H) the corresponding EXAFS spectra of R-Te-ACs@NC during one-day exposure to air; and (I) schematic diagram illustrating the deactivation and regeneration process.

In Figure 4A, during the 48-h long HER, the Te K-edge gradually shifts to higher energy, and the shape of the XANES curve changes, indicating structural reconstruction and changes in the oxidation state. Over time, the Te K-edge approaches the *E*_0_ of TeO_2_, and the fitted oxidation state of Te increases to 1.92 (Figure S21). The EXAFS data reveal a gradual increase in the intensity of Te–O bond pairs at 1.41 and 3.49 Å (Figure 4B), alongside a decrease in both the scattering-path intensity and coordination number for Te–Te bonding (Figure S22 and Table S4). These findings suggest progressive partial oxidation on the surface of Te-ACs@NC. To trace the chemical composition of Te-ACs@NC during *in situ* electrochemical reduction, a second time-scale resolution XAS was conducted (10s/spectrum). This method offers time-resolved information that aligns with the time scale of the electrochemical system, enabling maximum exploration of real-time changes in the catalyst’s composition and structure under realistic reaction conditions. *In situ* time-resolved XAS tracked the evolution of the Te K-edge over time (Figures 4C and S23). By monitoring the XAS spectra at 10 s interval, we accessed the real-time transition of the Te oxidation state. The Te K-edge gradually shifted to the lower energy region and was stabilized at 480 s. After 960 s, the Te absorption edge of Te-ACs@NC became practically identical to that of the Te foil. *In situ* time-resolved EXAFS (Figures 4D,E and S24) confirmed that the intensity of the Te–Te bond pair at 2.6 Å remained relatively stable during the first 480 s. However, the peaks at 1.41 and 3.49 Å, corresponding to Te–O bond pairs, showed a significant decline. This likely stems from the transformation of TeO_2_ to Te.

To quantitatively analyze the dynamic composition and oxidation state changes during electrochemical reduction, we fitted the *in situ* spectra using a linear combination of Te foil and TeO_2_, assuming that the measurements of reference samples accurately represent the 0 and +4 oxidation states. Using linear combination fitting (LCF), we calculated the ratio of Te and TeO_2_ species present at each time point (Figures 4F and S25). In the first 5 min, the proportion of Te^4+^ dropped from 33% to 5%, and further to 1% after the next 8 min. Subsequently, the proportion of Te^4+^ remained below 1%. The time-resolved XAS tests demonstrate that under *in situ* electrochemical reduction conditions, the surface Te oxides can vanish rapidly and be reduced to Te. Notably, XAS intensity and ICP-MS measurements indicate negligible Te dissolution or redeposition, confirming that the catalyst remains structurally intact during HER (Figure S26 and Table S5). Finally, we employed *in situ* XAS to examine the surface structure and composition changes of R-Te-ACs@NC during one-day exposure to air (Figure 4G,H). Over 24 h, we observed that the Te K-edge in XANES gradually shifted toward higher energy. Simultaneously, the peaks corresponding to the Te–O bond pairs in EXAFS increased significantly. Notably, after 24 h, the absorption edge of the R-Te-ACs@NC-air and the peaks corresponding to Te–O bond pairs showed no further changes (Figure S27). Clearly, atmospheric oxygen can partially oxidize R-Te-ACs@NC into a trapped state that is beneficial and achieves higher performance in the HER than a fully oxidized one.

Observations from *in situ* time-resolved XAS indicate that the deactivation and regeneration process of Te-ACs@NC can be divided into three stages (Figure 4I). First, Te-ACs@NC gradually loses catalytic activity upon prolonged HER operation as surface Te is stepwise oxidized to TeO_2_. Second, through *in situ* electrochemical reduction of deactivated Te-ACs@NC, surface oxygen is removed, reducing TeO_2_ back to Te. However, the catalytic activity of the Te catalyst is not fully restored, indicating that pure Te without oxidation does not achieve its desired high-performance state. Finally, the reduced catalyst stored in air undergoes spontaneous partial oxidation. XANES and EXAFS support the notion that the structure of the catalyst returns to a locally oxidized state. Simultaneously, the catalytic performance is optimized for Te-ACs@NC, indicating that localized oxygen on the cluster surface plays a performance-regulatory role in the HER activity of the Te active site.

### Theoretical Investigations on HER Activity

**Figure 5:**
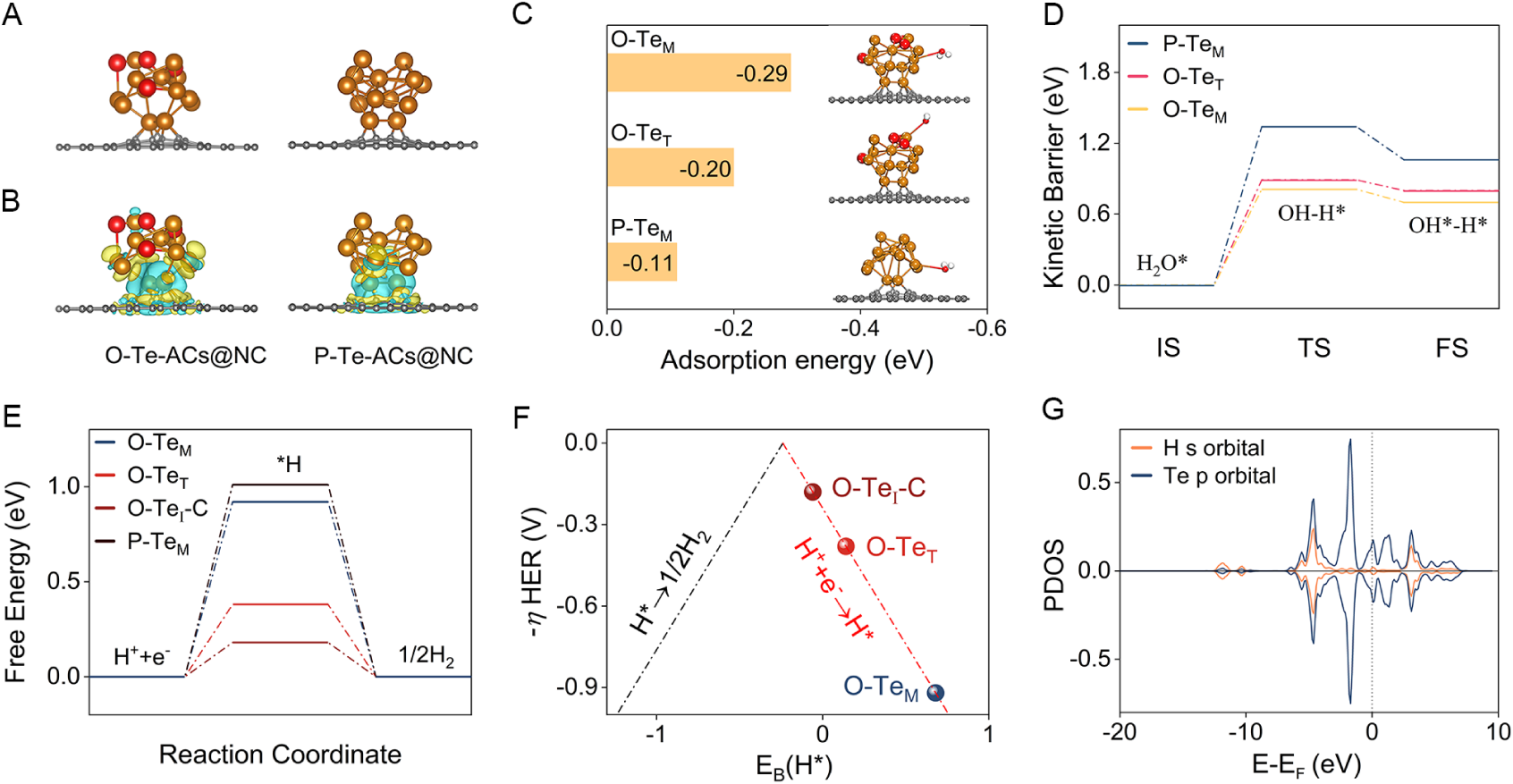
Theoretical calculation. (A) DFT-optimized crystal structures of O–Te-ACs@NC and P–Te-ACs@NC; (B) Differential charge density distributions corresponding to these structures, with yellow regions indicating charge accumulation and cyan regions showing charge depletion; (C) water adsorption energy at the active sites of various catalysts; (D) kinetic barriers for water dissociation at the active sites of different catalysts, with IS, TS, and FS representing the initial state, transition state, and final state, respectively; (E) calculated ΔGH* of the different active sites; (F) volcano plot of the theoretical overpotential for HER (ηHER) versus the binding energy of H atoms (*E*_B_) on Te or C atoms of various catalysts, compared to the standard hydrogen electrode; and ; (G) PDOS of the *p*-orbital of Te and the *s*-orbital of adsorbed H atom.

To theoretically understand the superior HER performance due to partial oxidation in Te-AC@NC, DFT calculations were performed. Using structural features, simplified models were created for locally oxidized Te-ACs@NC (termed O–Te-ACs@NC) and pristine Te-ACs@NC (termed P–Te-ACs@NC) (Figure 5A and Table S6). In the case of the O–Te-ACs@NC, a Te_13_ cluster, as a chosen model, undergoes partial oxidation with four oxygen atoms at its apex. Three types of surface Te atoms and one type of C serve as active sites (Figure S28), namely O–Te_T_, where Te is bonded to an oxygen atom at the cluster’s top; O–Te_M_, located in the middle of the cluster surface; and O–Te_I_ and O–Te_I_-C, at the cluster’s interface with the carbon matrix, directly connected to C/Te. In P–Te-ACs@NC, two surface atom types were identified: P–Te_M_ and P–Te_I_, which differ in their direct connections to carbon atoms. Differential charge density analysis (Figure 5B) revealed that in O–Te-ACs@NC, more charge transfer from O to Te atoms occurs, creating an electron-rich Te active site at O–Te_M_. Notably, unlike those of Te-NPs-1@NC and Te-NPs-2@NC, the level of localized oxidation in Te-ACs@NC does not increase over time. The higher oxidation state of O–Te_M_, due to its electron-rich nature, protects the Te cluster from further oxidation, consistent with XPS findings.

In alkaline HER, the overall reaction involves three key steps: water dissociation, formation of adsorbed hydrogen intermediates (H_2_O + e^–^ + * → H* + OH^–^), and hydrogen generation (H* + e^–^ → 1/2 H_2_). Favorable kinetics of water adsorption and dissociation are essential for the HER in alkaline electrolytes, directly influencing the HER activity. Initially, we examined the water adsorption process on the catalyst surface. Figure 5C shows that the calculated adsorption energy of H_2_O is −0.11 eV for P–Te_M_ and −0.20 eV for O–Te_T_. Upon introduction of oxygen, the binding of water molecules to O–Te_M_ is strengthened, as indicated by a reduced adsorption energy to −0.29 eV, highlighting the significant impact of local oxidation on water adsorption. Notably, due to steric hindrance, O–Te_I_ and P–Te_I_ are inefficient for water adsorption. For P–Te_M_, the kinetic energy barrier for water dissociation is relatively high at 1.34 eV, impeding OH–H bond cleavage in water to form *H intermediates (Figures 5D and S29). The energy barrier for water dissociation is significantly lower for O–Te_T_ at 0.89 eV, and further reduced to 0.81 eV for O–Te_M_, indicating enhanced HO–H bond cleavage and improved water dissociation on O–Te-ACs@NC due to surface oxidation. However, on O–Te_M_, *H preferentially adsorbs on the Te atom with a Gibbs free energy (ΔGH) of 0.92 eV (Figure 5E). This indicates significant proton trapping at Te, which limits hydrogen desorption. Conversely, the ΔGH for the C site is only 0.18 eV, with its η_HER_ near the volcano peak, indicating that O–Te_I_-C is more conducive to hydrogen production and balancing proton adsorption and desorption (Figure 5F). PDOS analyses of H-adsorbed C and Te atoms in the O–Te-ACs@NC provided insights into how the inherent electronic properties of Te and C affect H adsorption (Figures 5G and S30). The C orbital of the C atom is distanced from the Fermi level, in contrast to the Te *p* orbital, which shifts upward toward it. According to orbital theory, the C–H bond is significantly weaker than the Te–H bond, aligning well with ΔGH values.^40^ This suggests that H_2_ desorption is facilitated by H spillover from Te to the surface C site. H spillover also helps equalize the adsorption energy of reactive intermediates. This process not only accelerates H_2_O dissociation but also prevents excessive *H and *OH adsorption, which could inhibit active sites. As a result, it enhances the efficiency of the alkaline HER. Figure S31 summarizes the HER free energy values for the Te site (black line) and C site (red line) in the O–Te-ACs@NC via hydrogen spillover (Tables S7 and S8). With a ΔGH* of 0.18 eV, closer to zero than the Te-only pathway (0.38 eV), it highlights enhanced H_2_ production. Additionally, the free energy barrier for hydrogen formation is 0.92 eV for the Te-only pathway and 0.18 eV for the hydrogen spillover pathway via the Tafel step in the O–Te-ACs@NC, suggesting that the Heyrovsky step is more favorable than the Tafel process at its surface sites. In short, alkaline HER on the aqueous surface of the O–Te-ACs@NC involves H_2_O dissociation with strong H adsorption at the Te site, hydrogen spillover to the C site, and efficient H desorption at the C site. Local oxidation enhances water adsorption and lowers the energy barrier for water dissociation on Te-ACs@NC. The adjustable electronic structure of Te-ACs@NC allows for optimized adsorption energy of intermediates. Importantly, local oxidation on Te-ACs@NC facilitates hydrogen spillover from Te to C, with multisite effects and elemental synergy boosting electrocatalytic hydrogen evolution activity.

## Conclusion

We employed *in situ* electrochemical reduction to explore the reactivation of Te-ACs@NC deactivated by surface oxidation in prolonged HER operation. Our results verify that applying a reductive potential followed by aerobic storage enables the effective restoration of deactivated Te-ACs@NC in HER for reuse. As such, the HER catalytic activity remains well-preserved after multiple regenerations, proving the origin of long-life durability in the Te cluster catalytic system. *In situ* time-resolved XAS measurements crucially reveal that, under reduction potential, surface oxygen atoms on the deactivated Te-ACs@NC are gradually removed to reduce TeO_2_ to Te. The fully reduced Te-ACs@NC, with relatively lower activity for HER, is not at peak performance and is subsequently partially reoxidized in air to form the desirable highly active catalytic sites. DFT calculations suggest that partially oxidized Te-ACs@NC enhances water adsorption and lowers dissociation energy, playing a critical role in enhancing HER catalytic activity. Our findings pinpoint the power of *in situ* synchrotron analysis in unveiling the origin of high performance and the fundamentals of the fading mechanism for electrocatalytic systems, which can increase longevity.

## Methods

### Characterizations

SEM images were obtained using a JEOL JSM-7000F scanning electron microscope, and HAADF-STEM images were collected on a JEM ARM 200F operating at 200 kV with an aberration-corrected transmission electron microscope (Thermo Fisher Scientific Themis Z). Themis Z was used to acquire energy-dispersive X-ray spectroscopy (EDS) data for elemental mapping using a SuperX EDS detector. HAADF-STEM simulations were generated using the Dr. Probe software. X-ray diffraction (XRD) patterns were obtained by using the Cu Kα line (λ = 1.5418 Å). X-ray photoelectron spectroscopy (Thermo Fisher Escalab 250XI, USA) was used with a 150 W monochromatic Al–Kα X-ray source (*hυ* = 1486.6 eV) for measurements. The concentration of Te atoms was directly determined using an Agilent ICPOES730 inductively coupled plasma optical emission spectrometer (ICP-OES). Raman spectra were recorded using a Horiba LabRam HR 800 laser micro-Raman spectrometer with a 532-nm laser source.

### Synthesis of Quaternized Cellulose Nanofibrils

A mixture dispersion was prepared using a standard synthetic procedure by combining an aqueous solution of sodium hydroxide (NaOH, Thermo Scientific) with never-dried sulfite pulp (18 wt %, Nordic Paper). The concentrations of NaOH and pulp were 5 and 2 wt %, respectively. The dispersion was then agitated at 65 °C for 60 min. After adding a predetermined amount of glycidyltrimethylammonium chloride (TCI, 80 wt % in water), the dispersion was stirred at 65 °C for an additional 10 h. The reaction mixture was then neutralized with 0.5 M hydrochloric acid (37% solution in water, Thermo Scientific), filtered, and rinsed with deionized water until the ion concentration dropped below 1 ppm. A 1 wt % concentration of this chemically treated pulp in water was then processed through a microfluidizer (M-110EH, Micro Fluidics Ind., Newton, MA) with 200 and 100 μm chambers operating at 1600 bar of pressure at room temperature. The quaternized cellulose nanofibrils were obtained as a 1 wt % aqueous suspension.

### Synthesis of Te-ACs@NC and Its Counterparts

The typical synthesis of Te-based catalysts maintained mass ratios of pretreated quaternized cellulose nanofibrils (1%) to potassium tellurite (K_2_TeO_3_, Infinity Scientific, 99%) at 100:1, 100:2, and 100:10. These components were mixed with 50 mL of deionized water and stirred at 65 °C for 1 h to form a homogeneous Te@CNF mixture. The Te@CNF mixture was then solidified using liquid nitrogen and lyophilized on a freeze-dryer (Labconco Corporation, Kansas City, USA; FreeZone 2.5 L −84 °C Benchtop Freeze-Dryers). To eliminate unreacted K_2_TeO_3_, the Te@CNF aerogels were rinsed three times with deionized water and centrifuged at 12,000 rpm for 15 min. The Te@CNF was solidified again using liquid nitrogen and lyophilized once more with a freeze-dryer. The samples were pyrolyzed for 6 h at 900 °C under an Ar atmosphere to produce Te-NPs-1@NC, Te-NPs-2@NC, and Te-ACs@NC, respectively. For reference, a Te-free carbon sample, NC900, was produced using the same procedure, excluding the addition of K_2_TeO_3_. To remove oxygen from Te-ACs@NC, R-Te-ACs@NC was prepared *via in situ* electroreduction at −1.1 V vs RHE. Electrolysis was performed for 30 min for immediate characterization and testing.

### Electrochemical Measurements

All electrochemical measurements were conducted using a Bio-Logic SAS electrochemical workstation (VSP-300, France) in a three-electrode system at room temperature, with a graphite rod as the counter electrode and a saturated Ag/AgCl electrode as the reference electrode. The working electrode was prepared by ultrasonically dispersing 5 mg of catalyst and 50 μL of Nafion solution (5 wt %) in 1 mL of water/ethanol (1:1 vol/vol) to form a homogeneous ink. The ink (100 μL) was loaded onto carbon paper (1 cm^2^)) as the working electrode. For HER, cyclic voltammetry and linear sweep voltammetry were performed at a scan rate of 5 mV s^–1^ in 1.0 M KOH (pH 13.7). Long-term operational stability was evaluated by using chronoamperometric measurements. Electrochemical impedance spectroscopy (EIS) measurements were performed by applying an AC potential with a 10 mV amplitude over a frequency range from 100 kHz to 0.1 Hz. The electrochemical double-layer capacitance (*C*_dl_) was determined from the slope of the fitting line derived from CV curves. The electrochemical surface area (ECSA) was calculated using the equation: ECSA = *C*_dl_/Cs, where Cs, the specific capacitance, is generally in the range of 20–60 μF cm^–2^. In this work, a value of 40 μF cm^–2^ was used. All potentials were calibrated versus RHE using the equation:

E (RHE) = E (Ag/AgCl) + 0.197 V + 0.0591 × pH

The turnover frequency (TOF) of HER per Te site in the Te-ACs@NC electrocatalyst was calculated according to eq S1.
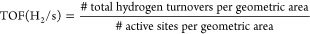
1

The total number of hydrogen turnovers per geometric area was calculated from the current density according to eq S2:
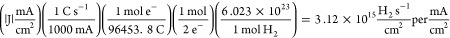
2

Since the exact hydrogen binding site is not known, we estimated the number of active sites as the number of surface sites on the Te cluster based on molar mass and mass loading. The Te cluster content was 1.7 wt %, and the mass loading was 0.5 mg cm^–2^. Similar approaches were previously used to estimate TOF for Co_1_/PCN, MoP, and CoP.^5,41,42^ The upper limit of active site density is 4.01 × 10^16^ Te sites per cm^2^.

Finally, the current density from the HER-LSV polarization curve can be converted into the TOF plot according to TOF = (3.12 × 10^15^/4.01 × 10^16^) × |J| = 0.077 |J|

Particularly, the current density of HER is 7.95 and 29.94 mA cm^–2^ at η50 and η100, respectively. Therefore, the TOF values of the Co_1_/PCN electrocatalyst were calculated to be TOF (50 mV) as 0.61 H_2_ s^–1^, and the TOF (100 mV) as 2.30 H_2_ s^–1^.

### XAS Measurements

XAS experiments were performed at the P64 beamline at PETRA III, DESY (Hamburg, Germany), and the Balder beamline at MAX IV Laboratory (Sweden). The absorption edge of a standard Te foil was used to calibrate the X-ray energy. For *ex situ* XAS measurements, the samples were pelletized into disks with BN to avoid self-absorption. *In situ* and time-resolved XAS measurements were conducted in fluorescence mode. The catalyst-coated ultrathin carbon paper was placed in a custom-built cell connected to a portable PalmSens4 electrochemical workstation. The cell was connected to the electrochemical workstation via electrical contact with a copper tape slip protruding from the side of the working cell. XAS spectra were recorded at different positions on the electrode to check the homogeneity of the catalyst. Spectra were first recorded on the dry catalyst, then in electrolyte solution at different working potentials, with at least three scans recorded at each potential. To obtain high-quality spectra, the time resolution of time-resolved XAS was 10 s, with a 6-s dead time between each measurement. Te K-edge theoretical XANES calculations were carried out using FEFF (version 9.6.4), with all structural models initially optimized by DFT. Each Te cluster model included a representative portion of the nitrogen-doped carbon matrix. Web ATOMS was employed to generate the necessary input files for FEFF, utilizing an optional CARD to specify computational parameters for the Te cluster system. The XANES simulations accounted for multiple coordination environments by calculating each site’s spectrum and then averaging them together (with no additional weighting factors). Distances are given in angstroms (Å) and energies in electronvolts (eV). LCF analysis was applied to the XANES region using Athena. TeO_2_ and Te foil were used for LCF analysis at the Te K edge as reference materials, with the fitting energy range referenced from *E*_0_: −15 to 40 eV. The goodness of fit was determined using the R-factor, as reported in Athena.

### Computational Details

All density functional theory (DFT) calculations were performed using the Vienna *ab initio* Simulation Package (VASP).^43^ The Perdew–Burke–Ernzerhof (PBE) functional was used to handle exchange-correlation interactions.^44^ A plane-wave basis set with a kinetic energy cutoff of 400 eV, an energy convergence criterion of 10^–4^ eV, a force convergence criterion of 0.02 eV Å^–1^, and a (2 × 2 × 1) Monkhorst–Pack k-point sampling were used for structure relaxation. A sufficiently large vacuum gap (>12 Å) was used to prevent interactions between neighboring periodic structures. H_2_ and H_2_O were calculated in 20 × 20 Å boxes using only the gamma point. Free energy diagrams for HER were calculated with reference to the computational hydrogen electrode.^45^ The free energy of the gas phase and adsorbed species was obtained using the following equation:
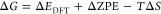


where *T* was set to 298.15 K, ΔZPE and *T*Δ*S* represent the changes in zero-point energy and entropy, respectively.

## Data Availability

The data supporting the findings of this study are available within the article and its Supporting Information files. All other relevant source data are available from the corresponding authors upon reasonable request.

## Supporting Information Available

SEM, TEM, HAADF-STEM, Element Mapping, XRD, XPS, Raman, XAFS, electrochemical tests, digital images, and additional methods , including photographs of the experimental setup (PDF)
