# Supplementary material for: In Situ Time-Resolved X-ray Absorption Spectroscopy Unveils Partial Re-Oxidation of Tellurium Cluster for Prolonged Lifespan in Hydrogen Evolution
Source: J Am Chem Soc. 2025 Apr 15;147(17):14359–68. doi: 10.1021/jacs.5c00167 (PMC12046551; doi:10.1021/jacs.5c00167)
Supplement: Supplementary file 1 — ja5c00167_si_001.pdf [file ja5c00167_si_001.pdf]

Supplementary Materials for

In situ Time-resolved X-ray Absorption Spectroscopy Unveils Partial Re-Oxidation of Tellurium Cluster for Prolonged Lifespan in Hydrogen Evolution

Kanglei Pang[1], Chang Long[2], Yu Zhang[1], Miao Zhang[1], Jian Chang[1], Yong-Lei Wang[1], Hao Zhang[1], Rongying Liu[1], Sadaf Saeedi Garakani[1], Özlem Uguz Neli[1], Jiayin Yuan[1]*

[1] Department of Chemistry, Stockholm University, Stockholm, 10691, Sweden.
[2] Institute of Fundamental and Frontier Sciences, University of Electronic Science and Technology of China, Chengdu, Sichuan, 611731, P. R. China.
*Corresponding authors. Email: jiayin.yuan@mmk.su.se

This file includes:

Figs. S1 to S24
Tables S1 to S4
References (1 to 3)

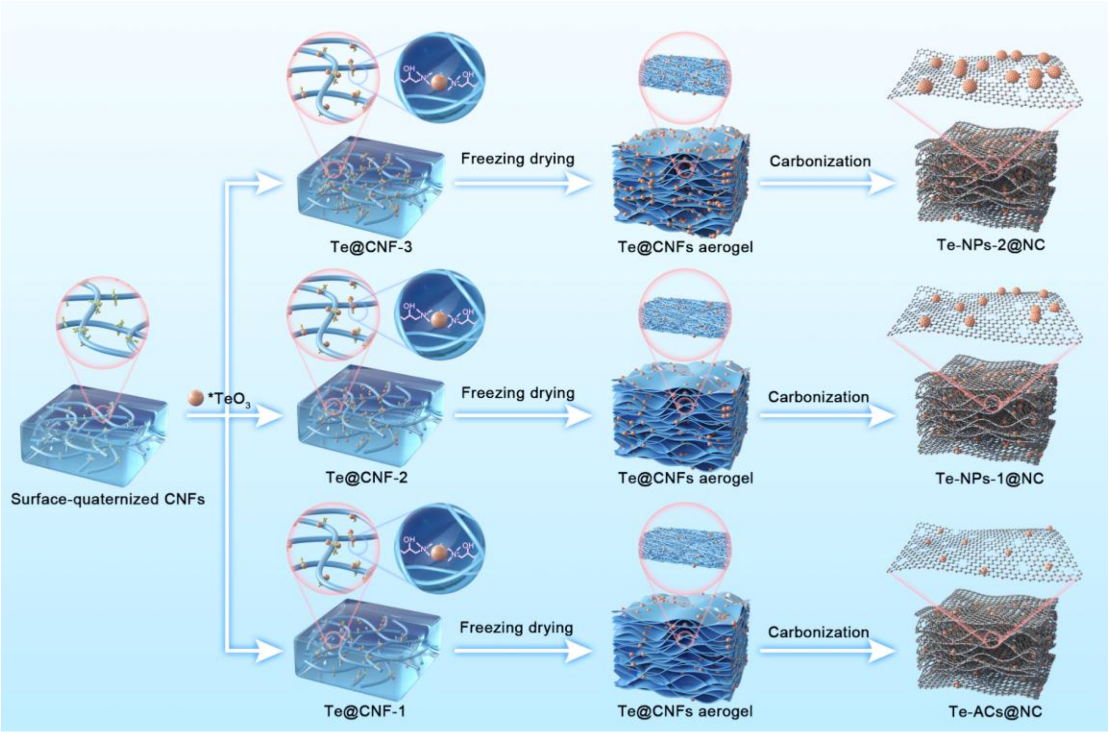

Figure S1. Preparation procedures of the Te-ACs@NC, Te-NPs-1@NC and Te-NPs-2@NC catalysts.

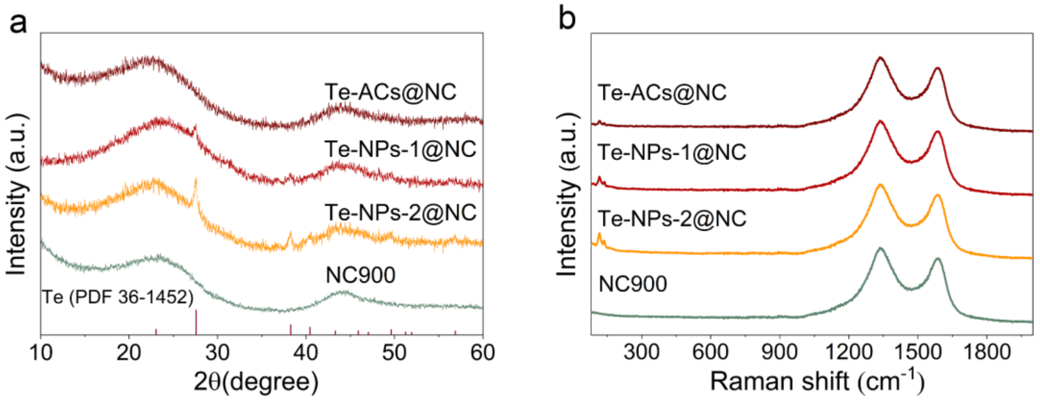

Figure S2. (a) XRD diagrams and (b) Raman spectra of Te-ACs@NC, Te-NPs-1@NC and Te-NPs-2@NC catalysts.

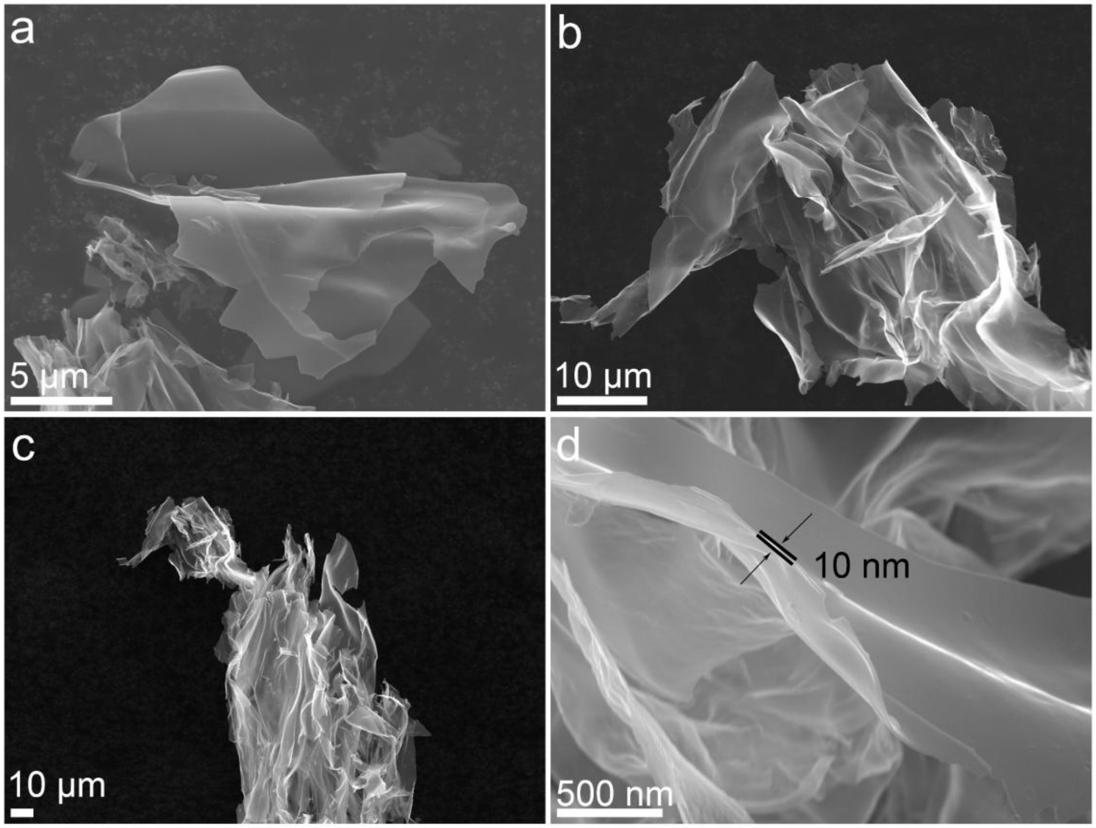

Figure S3. SEM images of (a) NC900, (b) Te-NPs-1@NC, (c) Te-NPs-2@NC and (d) its sliced view of a thin plate.

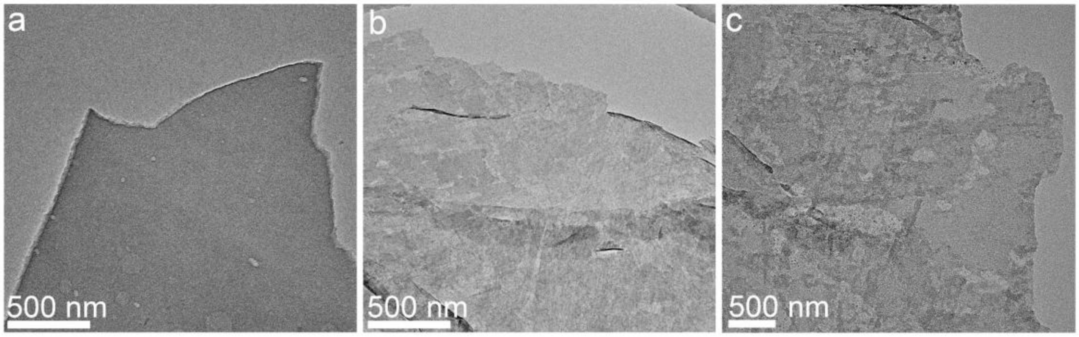

Figure S4. TEM images of (a) NC900, (b) Te-NPs-1@NC and (c) Te-NPs-2@NC.

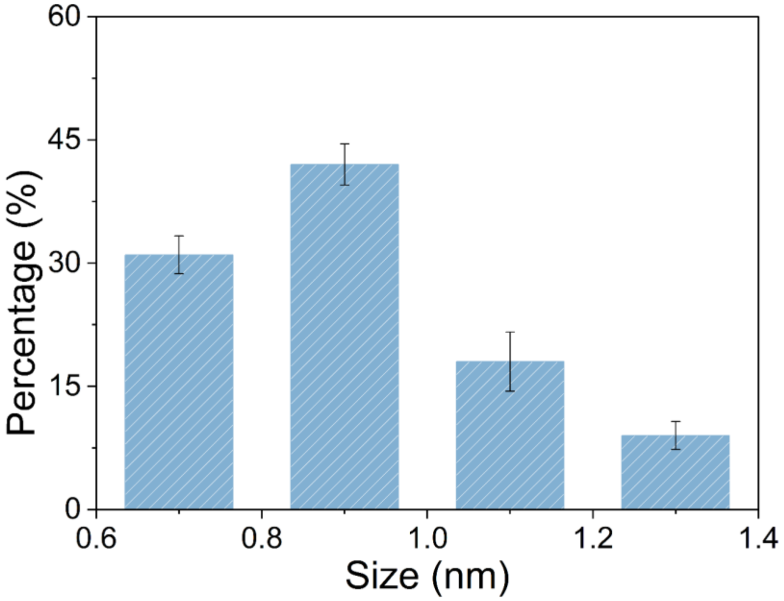

Figure S5. Size distribution histogram of Te clusters in Te-ACs@NC.

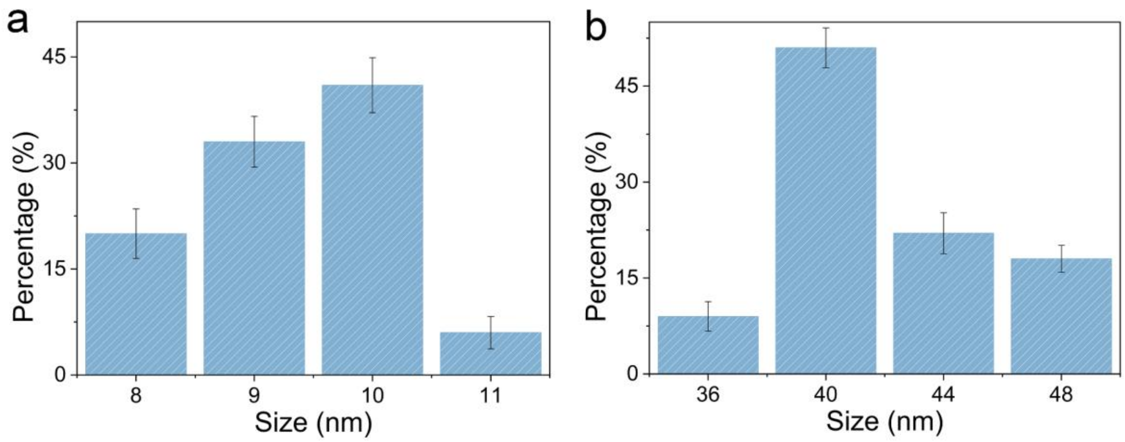

Figure S6. Size distribution histograms of Te NPs in (a) Te-NPs-1@NC and (b) Te-NPs-2@NC.

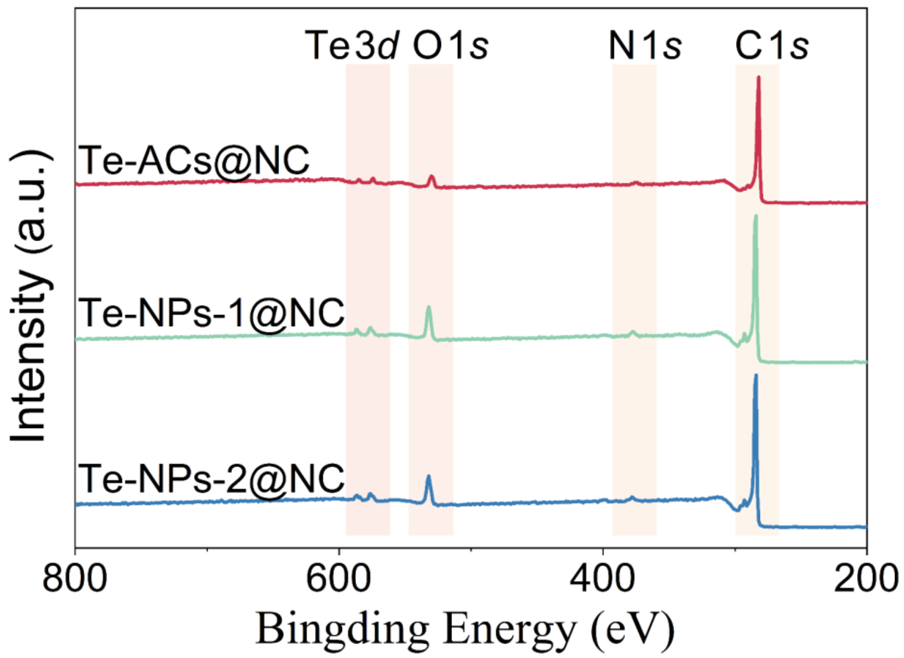

Figure S7. XPS full spectra of Te-ACs@NC, Te-NPs-1@NC and Te-NPs-2@NC.

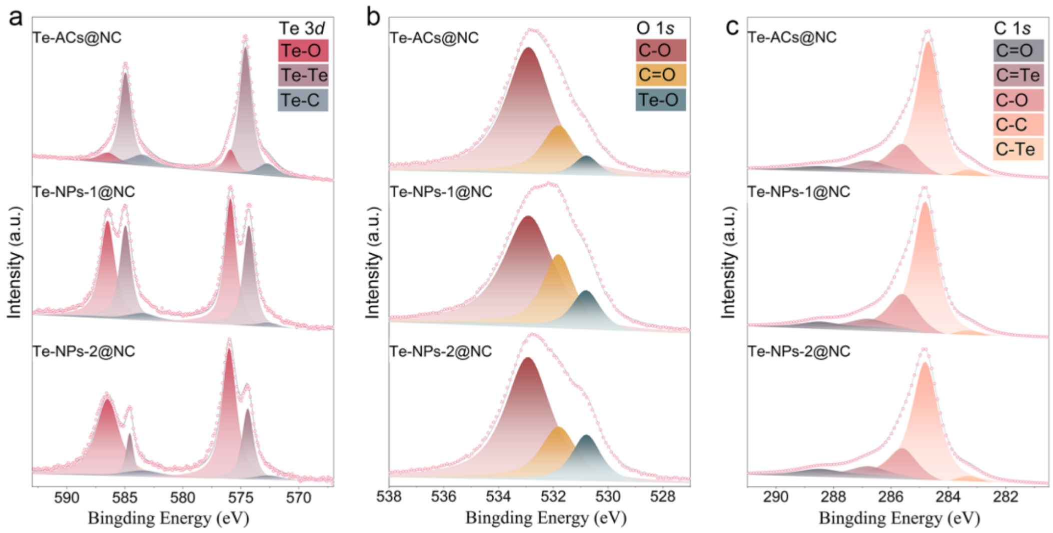

Figure S8. High-resolution XPS spectra of (a) Te 3d, (b) O 1s and (c) C 1s in Te-ACs@NC.

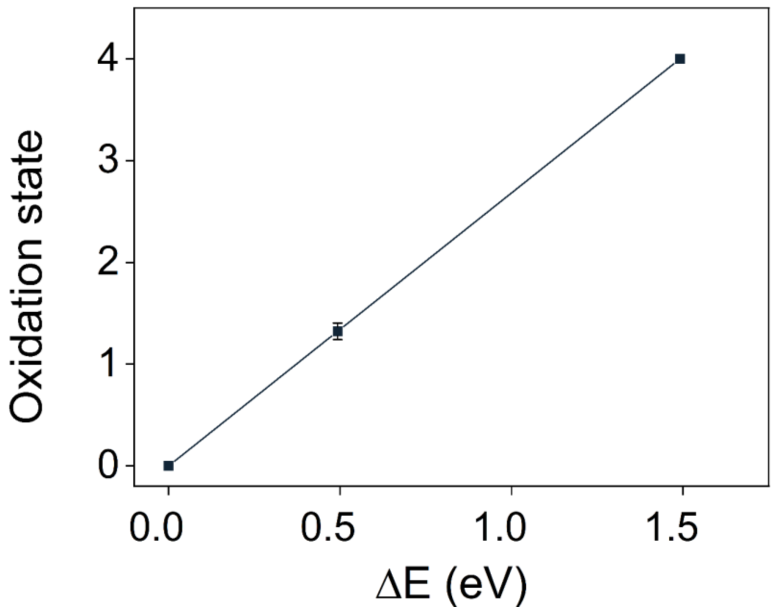

Figure S9. The fitted average oxidation states of Te-ACs@NC from XANES spectra.

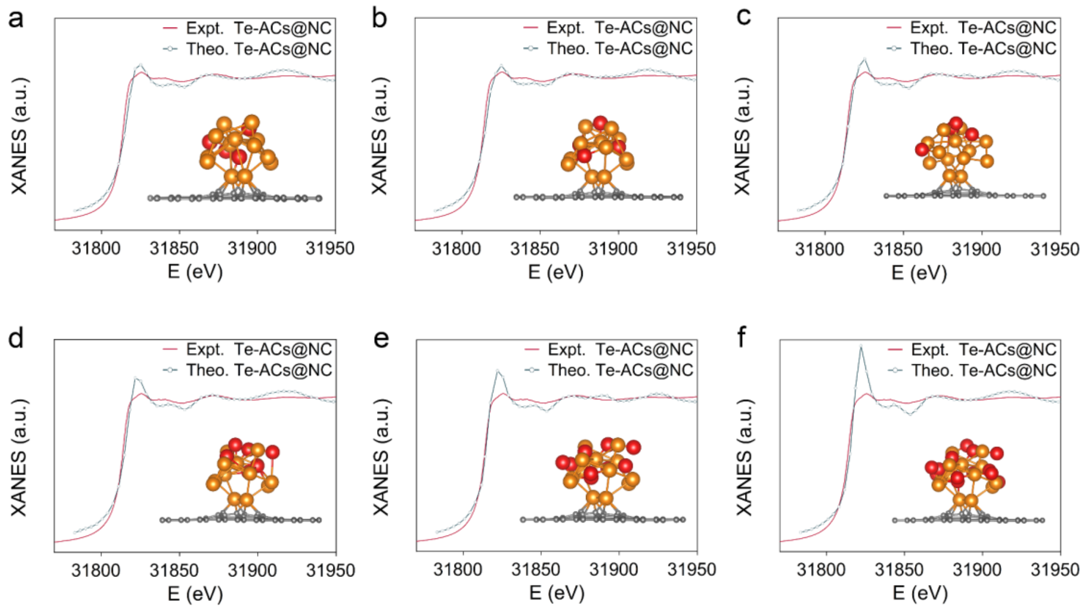

Figure S10. Comparison between the experimental Te K-edge XANES spectra and the theoretical spectra of different Te cluster model. The grayish-white, red, and yellow balls represent carbon (C), oxygen (O), and tellurium (Te) atoms, respectively.

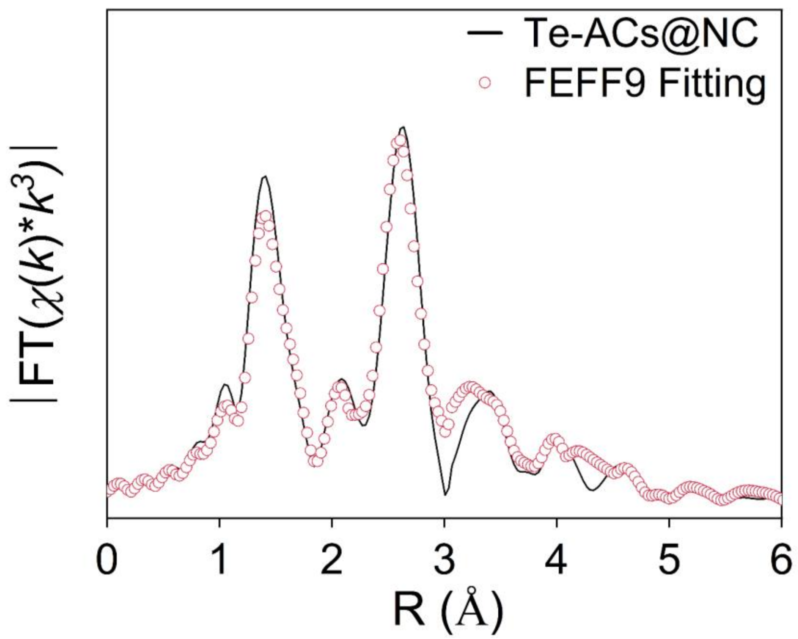

Figure S11. Comparison of experimental Te-ACs@NC FT EXAFS spectra with the theoretical spectra from FEFF9.

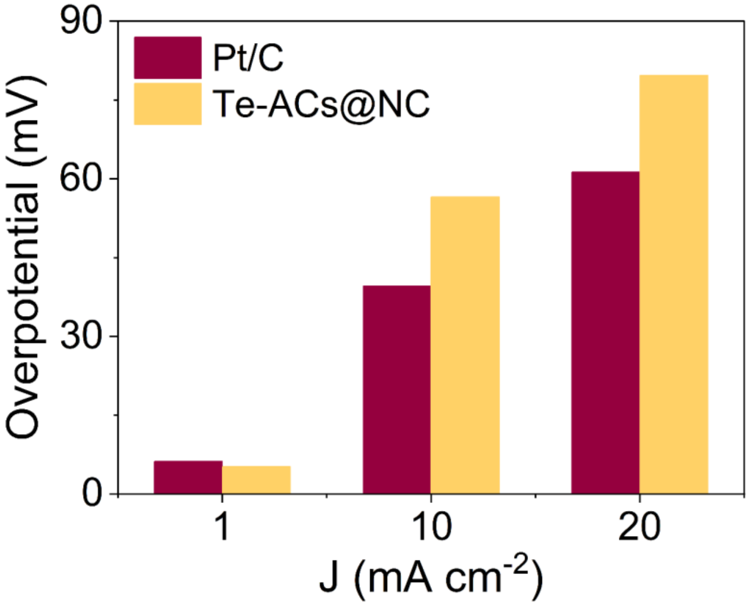

Figure S12. The overpotentials obtained from HER polarization curves at the current densities of 1, 10, and 20 mA cm^{-2} for Pt/C and Te-ACs@NC.

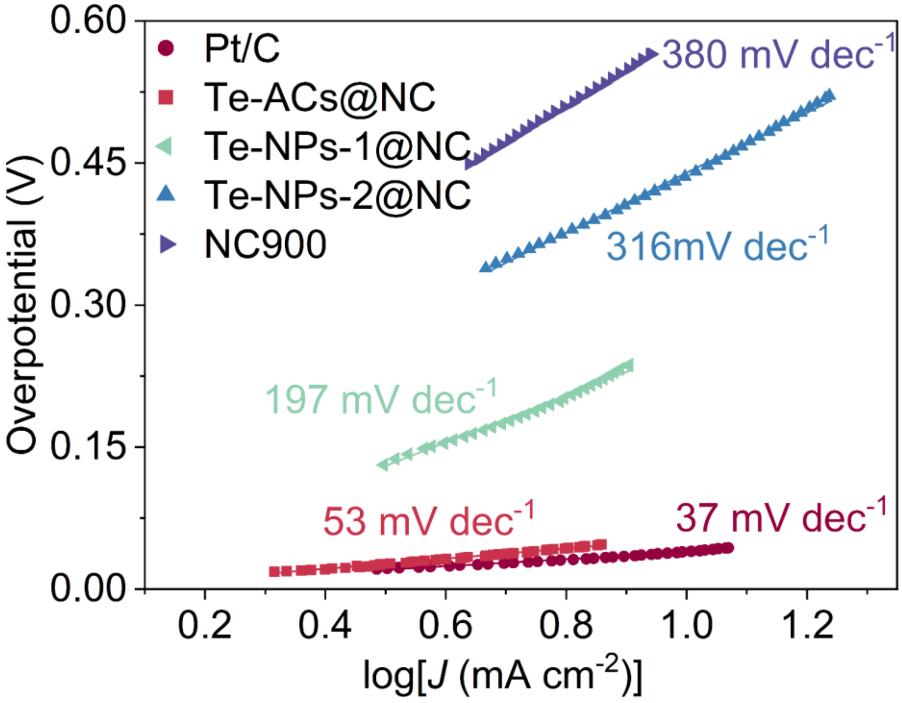

Figure S13. Representative Tafel slopes of Te-ACs@NC, Te-NPs-1@NC, Te-NPs-2@NC, NC900 and commercial Pt/C in 1.0 M KOH solution.

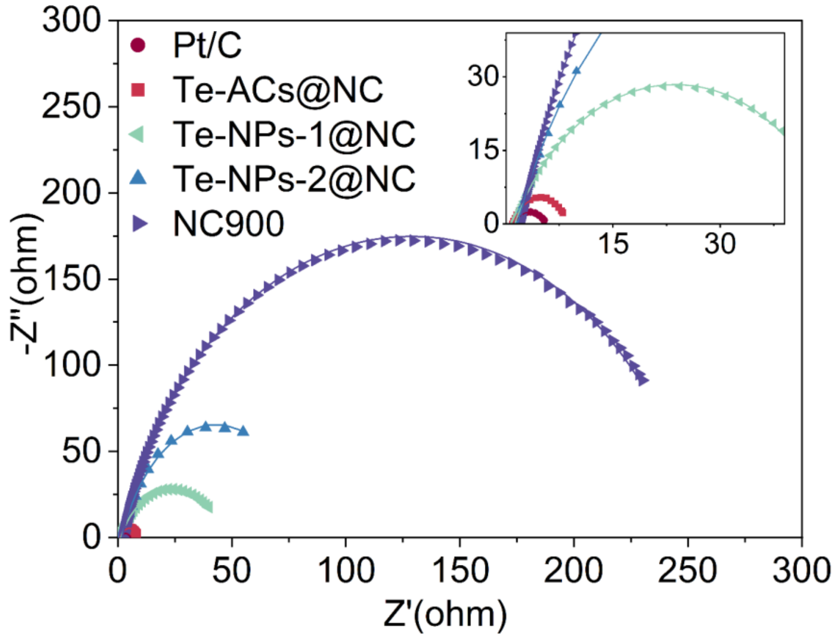

Figure S14. Representative Nyquist plots of Te-ACs@NC, Te-NPs-1@NC, Te-NPs-2@NC, NC900 and commercial Pt/C in 1.0 M KOH solution.

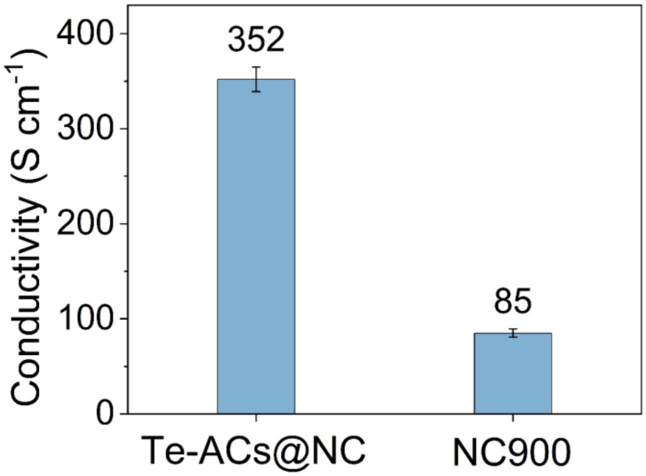

Figure S15. Electronic conductivity of Te-ACs@NC and NC900.

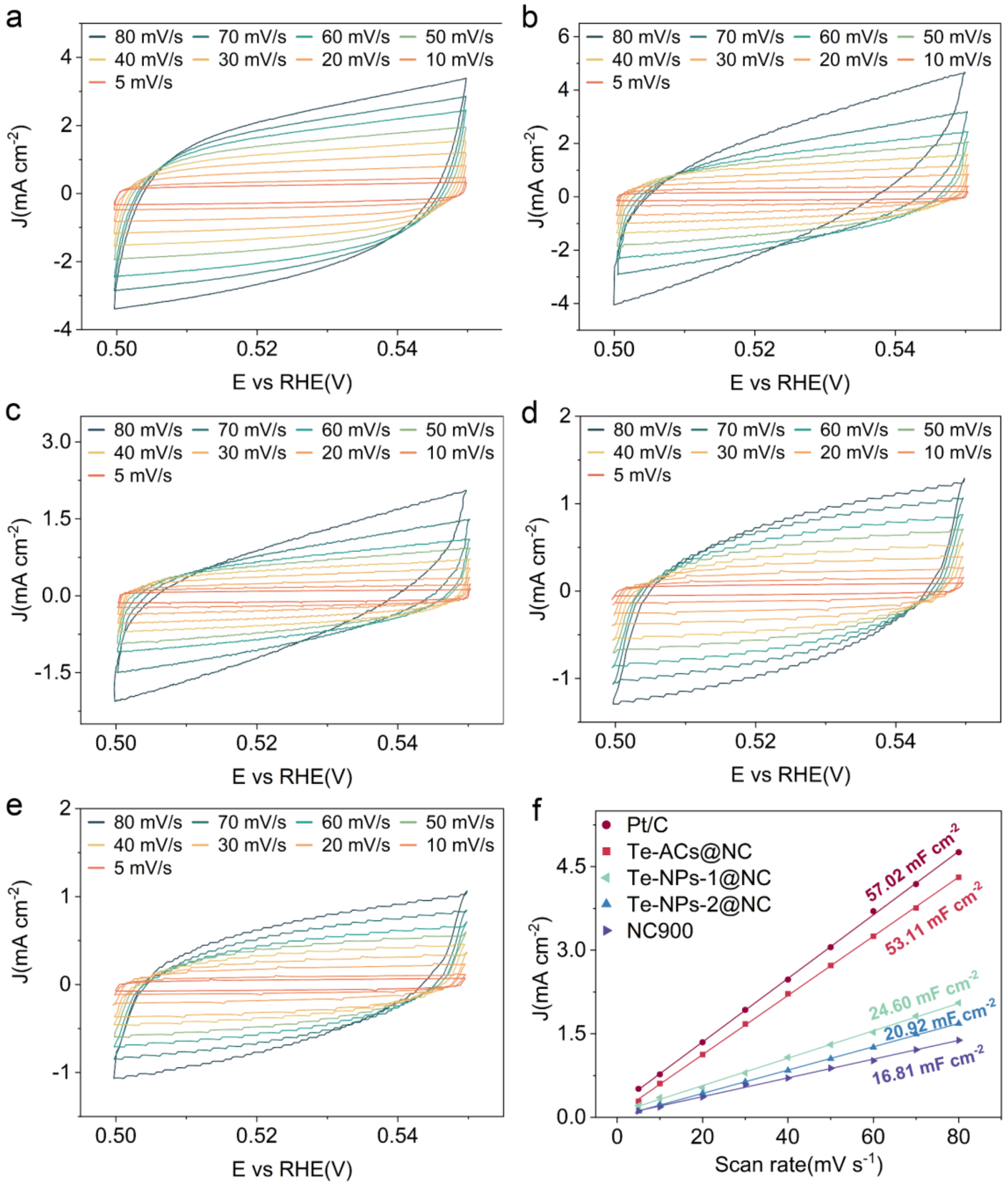

Figure S16. Charging currents measured in the non-faradaic potential from 0.50 V to 0.55 V (*vs* RHE) at different scan rates in 1 M KOH for (a) Pt@C, (b) Te-ACs@NC, (c)Te-NPs-1@NC, (d)Te-NPs-2@NC and (e)NC900; the double-layer capacitances of Te-ACs@NC, Te-NPs-1@NC, Te-NPs-2@NC, NC900 and commercial Pt/C.

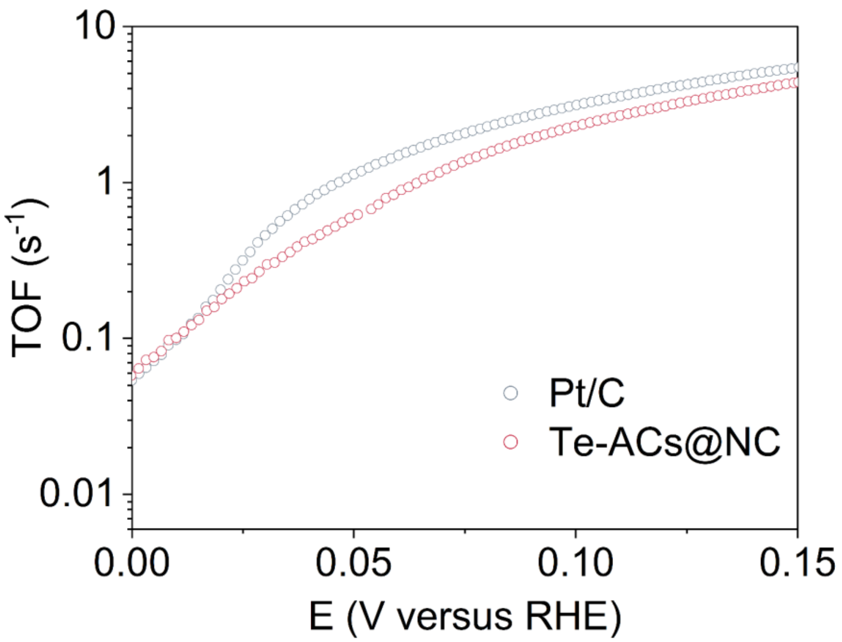

Figure S17. Correlation between TOF and HER overpotential for Te-ACs@NC and Pt/C electrocatalysts.

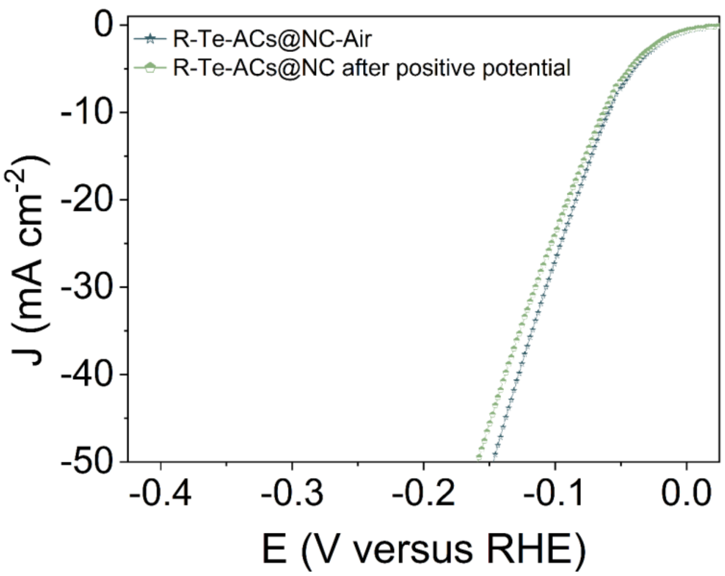

Figure S18. Representative HER polarization curves of R-Te-NPs@NC-Air and R-Te-NPs@NC after 30 mins positive potential treatment (0.8 V *vs* RHE) in 1.0 M aqueous KOH solution

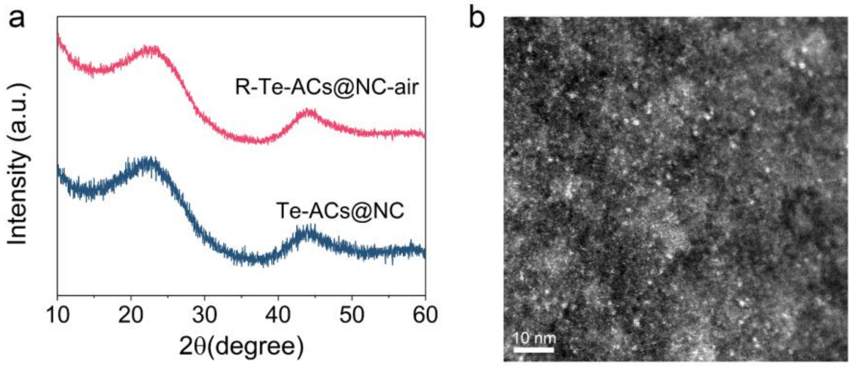

Figure S19. (a) XRD patterns and (b) HAADF-STEM image of R-Te-ACs@NC-air catalyst.

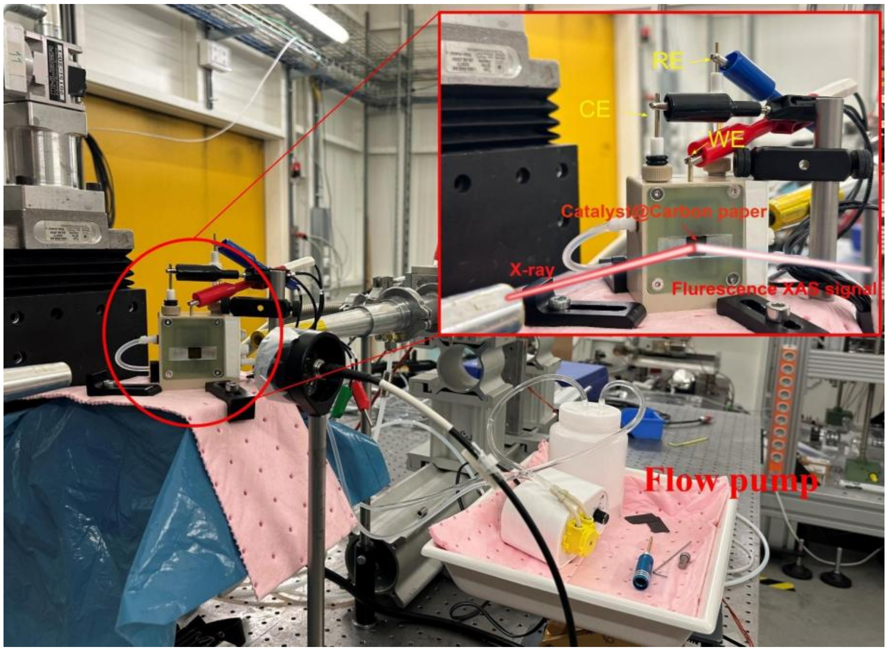

Figure S20. Photograph of *in situ* X-ray absorption spectroscopy measurement setup. The experiment was conducted at the P64 beamline with the support of PETRA III DESY. The X-ray induced fluorescence model was employed. CE denotes the counter electrode, WE represent the working electrode, and RE signifies the reference electrode. The working electrode was fixed by Kapton film and connected with conductive carbon paper.

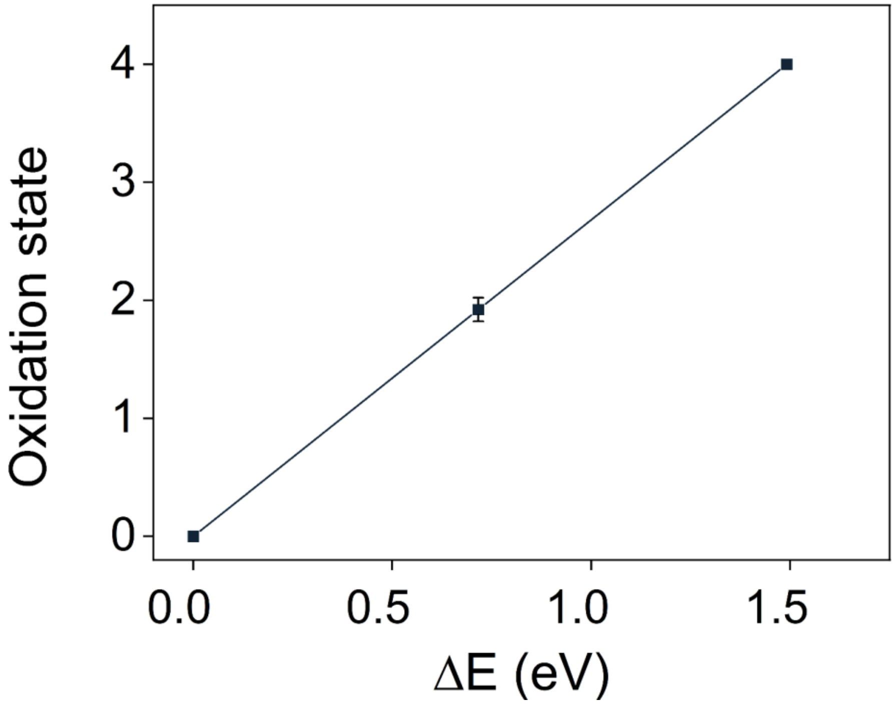

Figure S21. The fitted average oxidation states of Te-ACs@NC after 48 hs HER from XANES spectra.

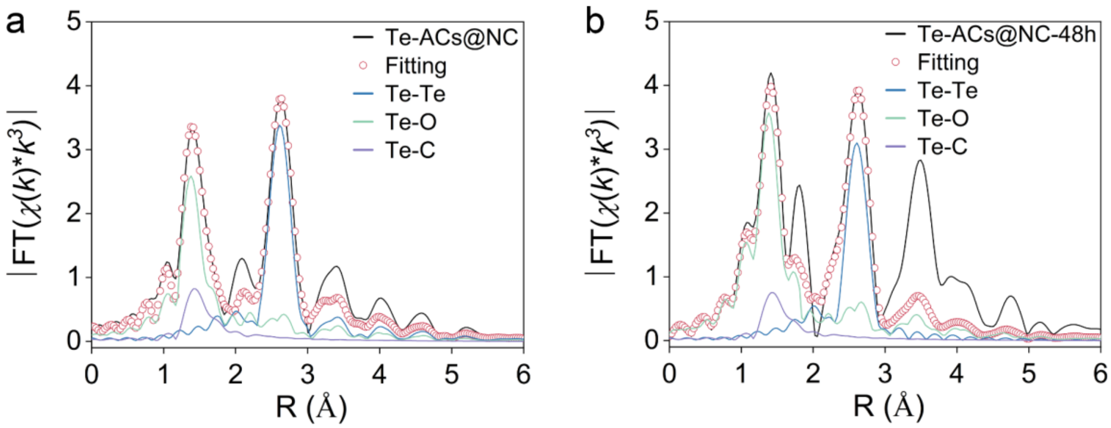

Figure S22. The fitting curves of k^3-weighted EXAFS spectra for (a) Te-ACs@NC and (b) Te-ACs@NC-48h.

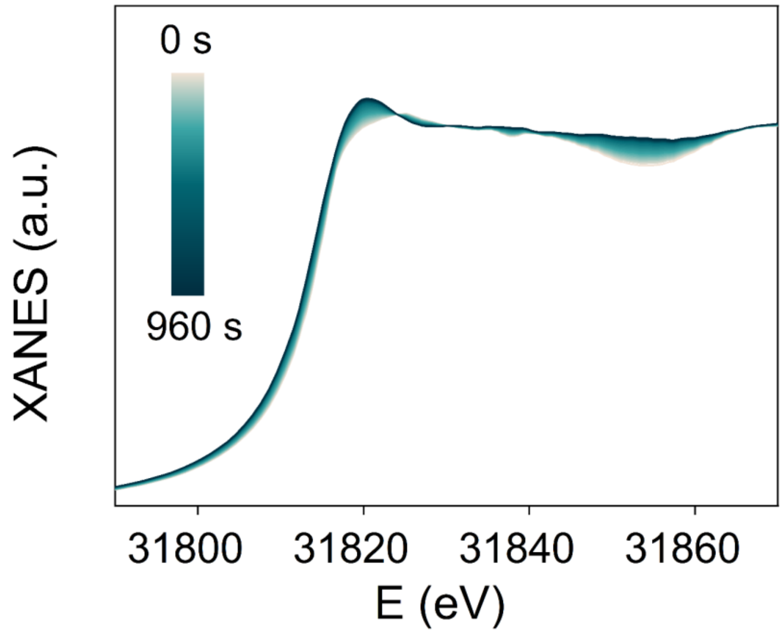

Figure S23. *In situ* time-resolved Te K-edge XANES spectra of Te-ACs@NC under *in situ* electrochemical reduction at -1.1 V vs. RHE.

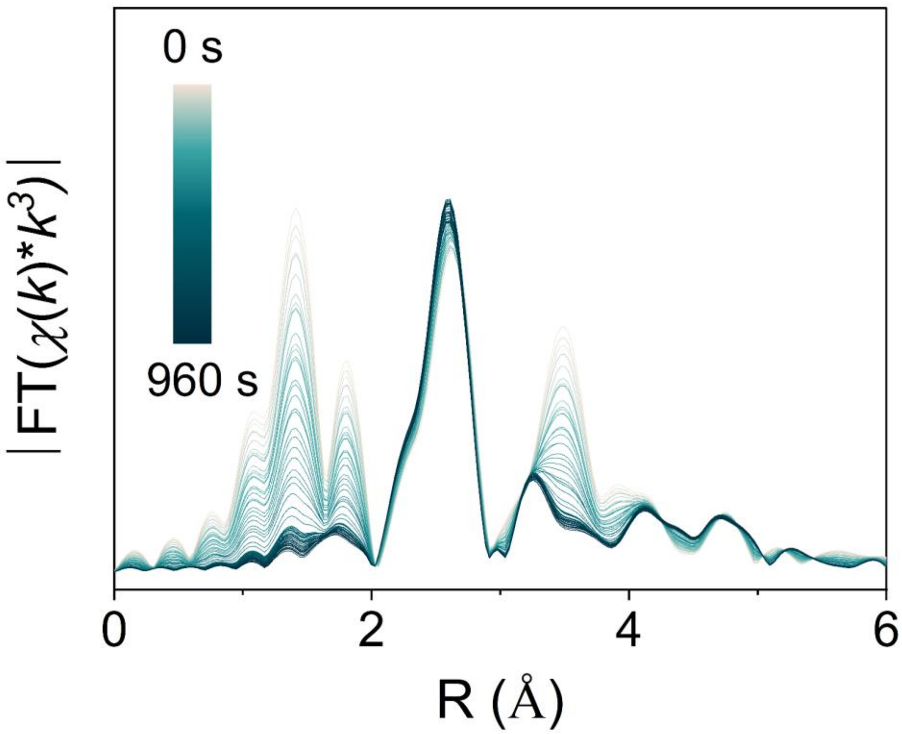

Figure S24. *In situ* time-resolved EXAFS spectra of Te-ACs@NC under *in situ* electrochemical reduction at -1.1 V vs. RHE.

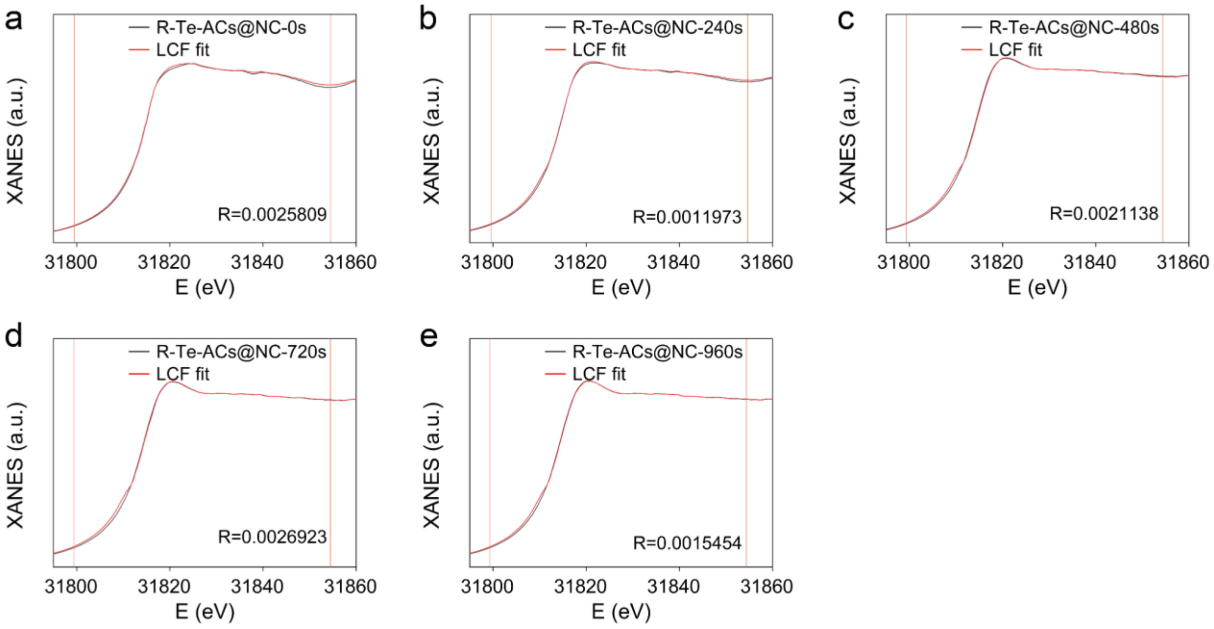

Figure S25. Linear combination fitting results of R-Te-ACs@NC under *in situ* electrochemical reduction at -1.1 V vs. RHE for (a) 0s, (b) 240s, (c) 480s, (d) 720s and (e) 960s. All R factors are inserted in their corresponding figures. Orange lines are inserted in figures to indicate the fitting range, which is from -15 eV to +40 eV based on E_0.

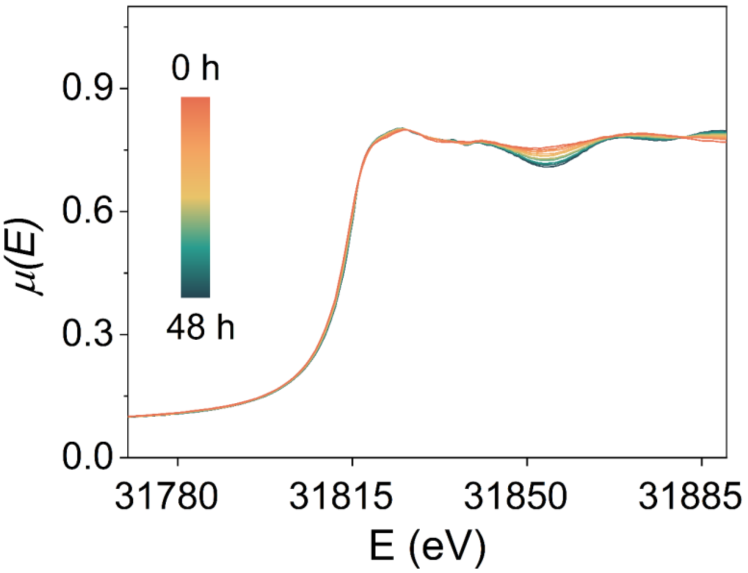

Figure S26. *In situ* Te K-edge XANES spectra recorded over 48 h of HER at 10 mA cm^{-2} in 1.0 M KOH.

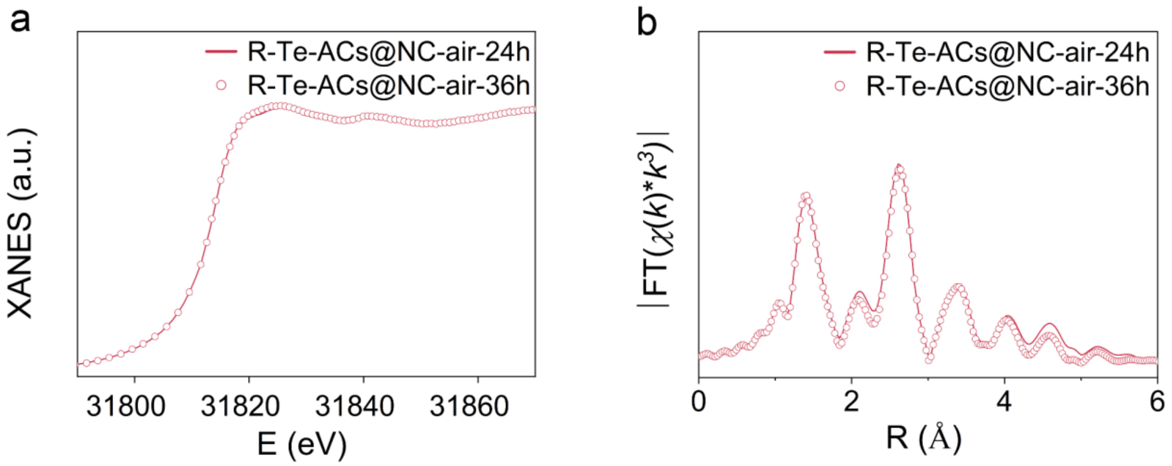

Figure S27. (a) Te K-edge XANES spectra and (b) the corresponding FT k^3-weighted EXAFS spectra of R-Te-ACs@NC after 24h and 36h of exposure to air.

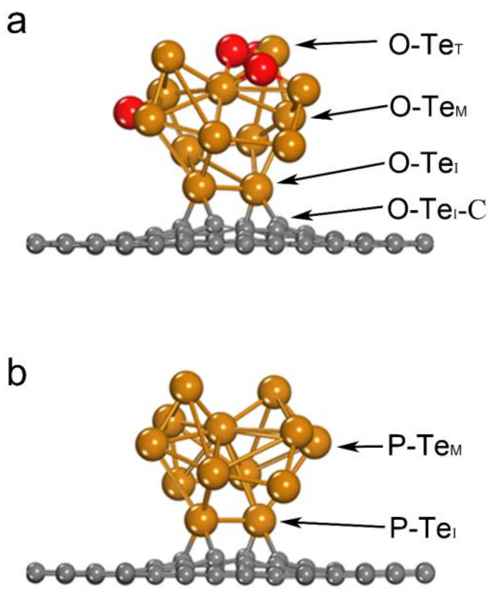

Figure S28. Different active sites for a) O-Te-ACs@NC and b) P-Te-ACs@NC.

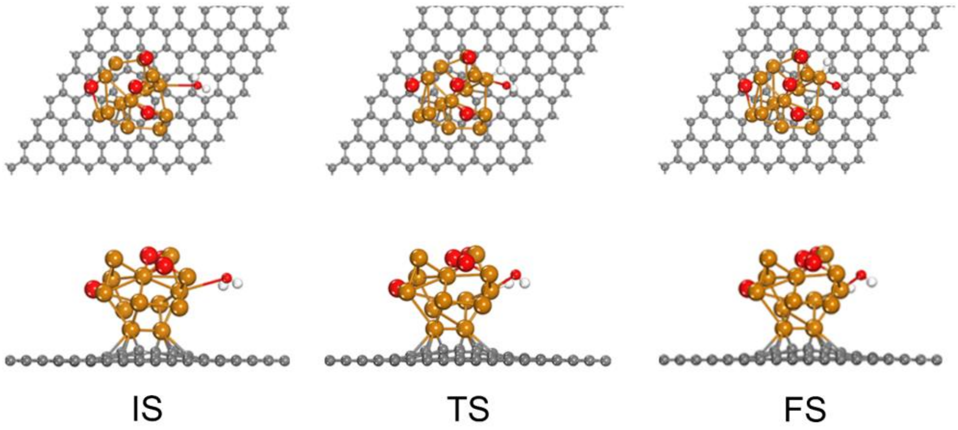

Figure S29. Top and side views of geometric structure about water dissociation on the O-Te_M active site. IS, TS and FS represent initial, transition state and final state, respectively.

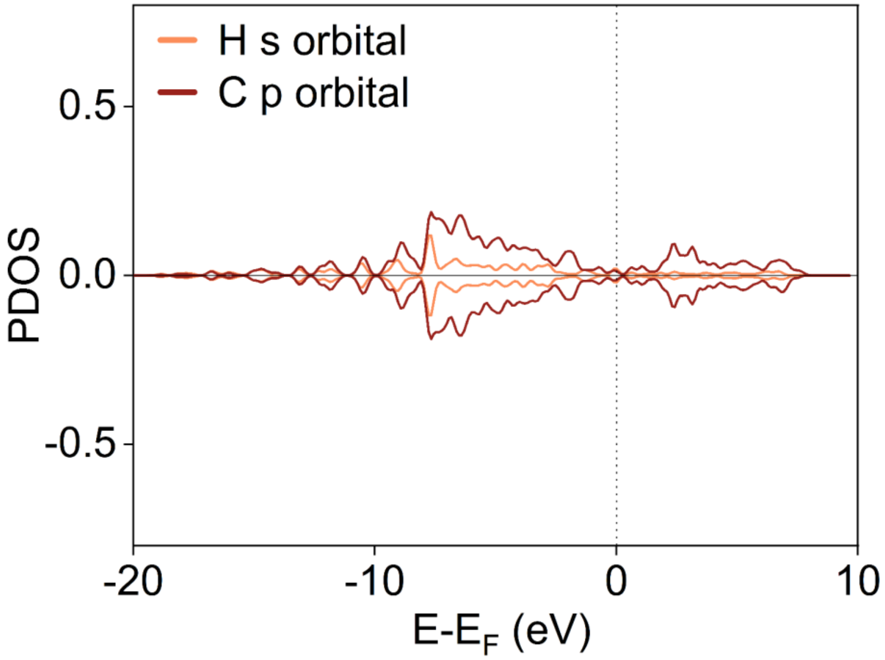

Figure S30. PDOS of the *p*-orbital of C and the *s*-orbital of adsorbed H atom.

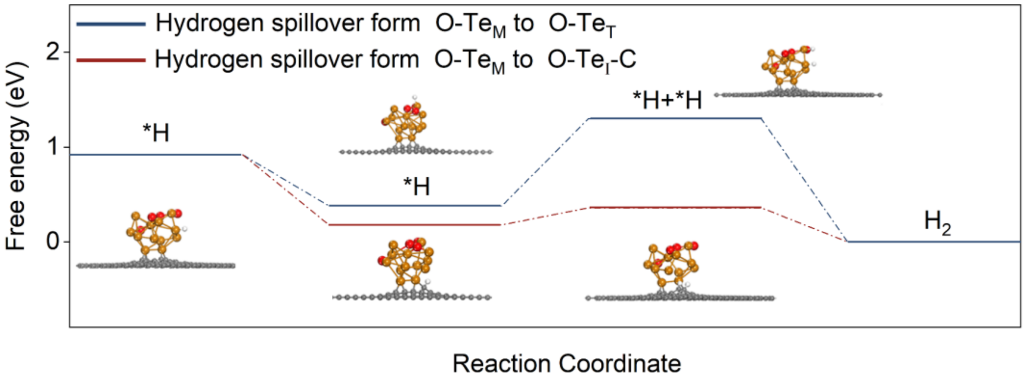

Figure S31. Gibbs free energy diagram for HER at Te atom and C atom through hydrogen spillover pathway for O-Te-ACs@NC.

Table S1. Elemental quantification determined by XPS for Te@CN catalysts.

Catalyst	C(at%)	N(at%)	O(at%)	Te(at%)
Te-ACs@NC	93.96	1.21	4.68	0.15
Te-NPs-1@NC	89.56	1.32	8.61	0.51
Te-NPs-2@NC	85.98	1.11	11.55	1.36
NC900	94.01	1.35	4.64	-

Table S2. Fitting results for Te 3d spectra for Te@NC catalysts.

Catalyst	Te-C (%)	Te-Te (%)	Te-O (%)
Te-ACs@NC	12.98	74.19	12.83
Te-NPs-1@NC	5.68	41.77	52.55
Te-NPs-2@NC	6.54	21.98	71.48

Table S3. Fitting results for Te 3*d* spectra for Te@NC catalysts after 30 days exposed to the air.

Catalyst	Te-C (%)	Te-Te (%)	Te-O (%)
Te-ACs@NC	13.05	73.43	13.52
Te-NPs-1@NC	6.23	34.60	59.17
Te-NPs-2@NC	6.89	17.85	75.26

Table S4. Structural parameters extracted from the quantitative Te K-edge EXAFS curve-fitting using ARTEMIS module of IFEFFIT.

Sample	Path	CN	R(Å)	σ^2 (10^{-3}Å^2)	ΔE_0(eV)	R factor
Te foil	Te-Te	2.2±0.1	2.85±.001	2.2±0.8	2.8±0.8	0.007
TeO_2	Te-O	1.9±0.1	1.85±0.02	2.8±1.5	5.2±1.3	0.031
	Te-O	7.2±0.2	2.53±0.03	**2.8**[a]		
	Te-Te	6.5±0.6	3.79±0.01	5.1±2.8		
Te-ACs@NC	Te-Te	2.2±0.1	2.87±0.03	3.5±1.6	6.5±1.2	0.028
	Te-O	1.7±0.3	1.86±0.02	3.7±1.5		
	Te-C	0.4±0.2	1.78±0.04	2.8±1.2		
Te-ACs@NC-48h	Te-Te	1.8±0.1	2.85±0.04	3.5±1.8	5.7±0.9	0.029
	Te-O	2.3±0.2	1.87±0.02	5.1±1.1		
	Te-C	0.4±0.1	1.74±0.02	2.5±1.3		
R-Te-ACs@NC	Te-Te	2.3±0.2	2.86±0.03	3.2±0.7	3.7±0.8	0.018
	Te-C	0.5±0.2	1.76±0.04	5.1±1.5		
R-Te-ACs@NC-air	Te-Te	2.2±0.2	2.88±0.05	4.0±1.2	8.6±1.5	0.026
	Te-O	1.9±0.5	1.87±0.06	3.9±1.7		
	Te-C	0.3±0.1	1.72±0.04	4.3±2.5		

S_0^2 was fixed as 0.92. CN: coordination number; R: bond length; σ^2: Debye-Waller factor; ΔE_0: inner potential shift. The fixed parameters are bolded.

The acquired EXAFS data were processed according to the standard procedures using the Athena and Artemis implemented in the IFEFFIT software packages[1]. The k^3-weighted χ(k) data were Fourier-transformed to real (R) space using a hanning windows (dk=1.0 Å^{-1}) to separate the EXAFS contributions from different coordination shells. The least-squares curve parameter fitting was performed using the ARTEMIS module of IFEFFIT software packages to obtain the quantitative structural parameters around central atoms[2]. Effective backscattering amplitudes F(k) and phase shifts $\Phi(k)$ of all fitting paths were calculated with the *ab initio* code FEFF8.0[3]. the difference between the number of independent data points and the number of fit parameters (The k-space range is from 2.0 to 12.0 Å^{-1}, R range is from 1.5−3.0 Å for Te foil, 1.0−3.9 Å for TeO_2, 1.0−3.0 Å for Te-ACs@NC, R-Te-ACs@NC and R-Te-ACs@NC-air). Shell-by-shell analysis was carried out to determine the local environment of Te in our samples and standards. [a] Due to the overlapping contributions in this spectral region there was significant correlation between the coordination number and the Debye−Waller factor of this shell, the latter was fixed to the value shown to stabilize the fit.

Table S5. Elemental quantification determined by ICP-MS for Te-ACs@NC based catalysts.

Sample	Te loading in catalysts (wt%)*	Te concentration in electrolyte solution (ng L^{-1})*
Te-ACs@NC	1.94 ± 0.02	1.36 ± 0.03
R-Te-ACs@NC	1.94± 0.02	1.35 ± 0.04
R-Te-ACs@NC-2	1.96 ± 0.03	1.33 ± 0.01
R-Te-ACs@NC-6	1.94 ± 0.06	1.36 ± 0.01
R-Te-ACs@NC-10	1.93 ± 0.01	1.34 ± 0.05
Te-ACs@NC-1h (under HER reaction)	1.94 ± 0.05	1.33 ± 0.03
Te-ACs@NC-12h (under HER reaction)	1.95 ± 0.01	1.32 ± 0.06
Te-ACs@NC-24h (under HER reaction)	1.92 ± 0.05	1.36 ± 0.01
Te-ACs@NC-48h (under HER reaction)	1.93 ± 0.01	1.36 ± 0.04
R-Te-ACs@NC-60s (under reduction)	1.93 ± 0.06	1.34 ± 0.06
R-Te-ACs@NC-240s (under reduction)	1.95 ± 0.06	1.35 ± 0.03
R-Te-ACs@NC-720s (under reduction)	1.92 ± 0.05	1.33 ± 0.02
R-Te-ACs@NC-960s (under reduction)	1.92 ± 0.03	1.32 ± 0.03

*Mean from three tests

Table S6. Bader Charge Analysis for O-Te-ACs@NC (locally oxidized) and P-Te-ACs@NC (without being locally oxidized).

O-Te-ACs@NC		P-Te-ACs@NC	
Element	Charge (a.u.)	Element	Charge (a.u.)
Te	4.7814	Te	5.7966
Te	5.2257	Te	6.0106
Te	5.9352	Te	6.0168
Te	6.0667	Te	6.1363
Te	6.1351	Te	5.9952
Te	6.0674	Te	5.9882
Te	4.3056	Te	6.0223
Te	5.0578	Te	6.0456
Te	5.8093	Te	6.1073
Te	4.9015	Te	4.8791
Te	4.8246	Te	4.8987
Te	5.9646	Te	6.109
Te	5.9638	Te	5.933
O	7.2485		
O	7.375		
O	7.3318		
O	7.1328		

Table S7. Adsorption energy change (ΔE), zero-point energy change (ΔZPE), entropy change (TΔS) where T =298K and relative free energy change (ΔG) of hydrogen spillover from Te_2 toTe_1.

Species	ΔE(eV)	ΔZPE (eV)	TΔS (eV)	ΔG (eV)
*H	-2.69	0.18	0.01	0.92
*H	-3.22	0.18	0.02	0.38
*H + *H	-2.69	0.18	0.01	1.30

Table S8. Adsorption energy change (ΔE), zero-point energy change (ΔZPE), entropy change (TΔS) where T =298K and relative free energy change (ΔG) of hydrogen spillover from Te_2 to C.

Species	ΔE(eV)	ΔZPE (eV)	TΔS (eV)	ΔG (eV)
*H	-2.69	0.18	0.01	0.92
*H	-3.44	0.19	0.02	0.18
*H + *H	-3.44	0.18	0.01	0.36

References

1. Newville, M. (2001). IFEFFIT : interactive XAFS analysis and FEFF fitting. J Synchrotron Radiat *8*, 322-324. doi:10.1107/S0909049500016964.

2. Ravel, B., and Newville, M. (2005). ATHENA, ARTEMIS, HEPHAESTUS: data analysis for X-ray absorption spectroscopy using IFEFFIT. J Synchrotron Radiat *12*, 537-541. doi:10.1107/S0909049505012719.

3. Ankudinov, A.L., Ravel, B., Rehr, J.J., and Conradson, S.D. (1998). Real-space multiple-scattering calculation and interpretation of x-ray-absorption near-edge structure. Phys. Rev. B *58*, 7565-7576. 10.1103/PhysRevB.58.7565.
